# Point-based method for measuring the phenotypic data of channel catfish (*Ictalurus punctatus*)

**DOI:** 10.1371/journal.pone.0324158

**Published:** 2025-06-05

**Authors:** Xiujun Zhang, Su Fang, Yuanbo Li, Xiaohui Chen, Fuyong Huang

**Affiliations:** 1 School of Applied Engineering, Zhejiang Business College, Hangzhou, Zhejiang, China; 2 School of Electronic Information Engineering, Zhejiang University, Hangzhou, Zhejiang, China; 3 Freshwater Fisheries Research Institute of Jiangsu Province, Nanjing, Jiangsu, China; 4 Zhejiang Academy of Agricultural Sciences, Hangzhou, Zhejiang, China; Penn State University, UNITED STATES OF AMERICA

## Abstract

In industrial societies, most fishery research institutes collect the phenotypic data of fish manually, which is time-consuming, labor-intensive, error-prone, and results in incomplete data. Considering their stress reaction and the natural body extension to collect the phenotypic data of fish quickly and accurately, channel catfish was used as the research subject and a deep-learning-based method was developed to explore their phenotypic data, i.e., body length, full length, head length, body height, tail handle width, tail handle height, and body thickness. First, this study applied two cameras and another device built into an image acquisition system to obtain images of fish in the water. We then adopted an Hourglass module network to position nine and ten key points on the top and side view images, building two key point fish skeletons. Finally, 3D coordinate transformation and scale parameters were employed to obtain the phenotypic data. Compared with the ground truth of the phenotypic fish data, our study achieved a 3.7% average relative error in terms of the full length, and an average 9.6% relative error for all seven types of phenotypic data applied. Furthermore, the average time required for the image processing measurements was approximately 1s.

## 1. Introduction

In 1984, Channel Catfish (*Ictalurus punctatus*) was introduced to China from the US [1]. Due to its desirable traits for commercial cultivation, the cultivation area and total production of channel catfish in China increased significantly, reaching 248,000 metric tons and generating over 50 million US dollars in export income in 2014 [2]. Obtaining fish phenotypic data can help providing management advice and beneficial the economic growth of fishery enterprises. Furthermore, phenotypic data can be utilized for body-size-based sales strategies, enabling maximum economic returns by determining optimal harvesting times [3]. In addition to these advantages, phenotypic data serve as an indispensable reference in fishery breeding research, providing critical insights for scientific studies. Furthermore, phenotypic data also plays a significant role in fields such as fish taxonomy and systematics, as well as fish biology and ecology [4,5]. However, a traditional manual measurement method is time-consuming, labor-intensive, and subjective. Moreover, out-of-water measurements may hurt the measured fish. Laser measurements are expensive and impractical, and thus vision-based measurements have been considered. Computer vision is a non-destructive [6], fast [7], economic [8], consistent [9], objective, repeatable [10], quantitative [11], relatively effective, and powerful tool [12,13]. The application of computer vision to live-fish measurements is a challenging task. Owing to the high efficiency of computer vision measurement technology, this method is becoming increasingly popular [14]. Over the years, numerous researchers have been exploring how to obtain phenotypic data on fishes from digital images more accurately and efficiently. Traditional phenotypic data measurement methods are mostly out-of-water measurements, which can be divided into two categories: linear and non-linear. Recent studies are summarized as follows.

### 1.1 Linear measurement

When the fish’s body is approximately a straight line, the length of the body is equal to the length of the line segment in the image. The fish length can be calculated based on the relation between the line segment in the image and the image scale parameters [11]. The image scale parameters can be obtained through a square on the color plate [15]. The problem is how to obtain the line segment length on the image. Hsieh et al. [11] employed gradient-based edge detection combined with the Hough transform to identify the head and tail positions of tuna species, such as Bigeye Tuna (*Thunnus obesus*) and Albacore (*Thunnus alalunga*), by analyzing both sides of the color plate grid. In addition, Balaban et al. [16] used the bounding box to determine the width and length of the fish simultaneously, this method is only suitable for measuring the length of fish with straight bodies, and the categories of measured phenotypic data are relatively limited. To avoid interference from the external environment, Abdullah et al. [17] used computer vision technology and curvature information to extract the length parameters of the fish. Although this method can calculate full-length information, it relies on traditional image processing techniques and optical formulas, requiring a five-step calculation process that is cumbersome. Nelson et al. [18] developed a method for collecting fish length data using Electronic Monitoring (EM) systems. In this method, haddock (*Melanogrammus aeglefinus*) is placed horizontally on a measuring board during the measurement process, but only the length dimensions of the fish can be output.

### 1.2 Non-linear measurement

There is a large deviation in the fish length measurement based on linear measurement, many current studies on fish phenotypic data measurement employ nonlinear measurement methods. One of the methods is estimating the length by key points. Strachan [7] used the key point method to measure the length of the Haddock (*Melanogrammus aeglefinus*) on the conveyor. This study was based on the keypoint method to measure the length of the haddock on a conveyor belt. Under ideal constraint conditions, the measurement error was less than 1%. However, if the fish’s posture was random, the error increased to 3%. The measurement method proposed in this paper can only be measured out of water and has strict requirements for the fish’s posture. White et al. [19] used a binarized image to locate the direction of the fish’s body and cut the binarized body equally to obtain the midpoint through the perpendicular of this direction. The accuracy of this method is high, but if the angle of the fishtail is large, the final measurement error increases significantly. In other methods, Romero et al. [20] proposed to use of a third-order polynomial equation to fit the centerline of the rainbow trout’s body contour and then combine the Mahalanobis distance with a threshold to classify the fish. Miranda et al. [21] proposed to use the ratio of pixel changes between each frame of video images to determine the rainbow trout entering a specific shooting area, and then used a third-order polynomial equation to fit the centerline of the fish body contour to determine the length.

The current methods for measuring fish length are highly complex. However, they are prone to significant measurement errors when the fish curl their bodies. At the same time, the processes are cumbersome, and most methods are out-of-water measurements, which can cause a high-stress reaction.

### 1.3 Deep learning

With the development of the BP (backpropagation algorithm) [22], deep learning has developed rapidly, and CNN (convolutional neural network) [23] is being widely used in various image application fields. Tseng et al. [24] proposed to use a convolutional neural network to detect the positions of fish heads, fish tails, color plates, and then combined traditional image processing methods to calculate the actual value of the fish length. The detection of this method is more complicated, and the position of fish head and fishtail points is susceptible to environmental interference. Monkman et al. [25] proposed using R-CNN to detect the pre-examination position and the area of the color plate. They used the length of the largest detection bounding box as the predicted width of the fish’s body, combined with an ArUco marker [26] to predict the final length value. The positioning accuracy of the bounding box limits this method. Wang et al. [27] used image segmentation methods to measure body length and height of Large Yellow Croaker (*Larimichthys crocea*), with an error of less than 4%. The above three measurement methods based on deep learning are all performed out of water. To measure fish in water, an underwater stereo vision system was used to complete the measurement of fish body length [28,29].

From the above research, we can see that the number of phenotypic data measured by all research methods is small, and deep learning can avoid the artificial design of complex measurement algorithms. Based on this discovery, a key point positioning method based on deep learning is considered the preferred method in this study. The Hourglass Network is a model proposed by Newell et al. [30]. The cascaded hourglass network module achieves precise positioning of human key points. Compared to other methods, this deep network model based on an Hourglass Network module has a concise and easy-to-expand structure, showing an accurate and efficient performance. This study proposes a key point location algorithm based on the Hourglass Network, combining 3D transformation and scale parameters to obtain phenotypic data.

The main contributions of the paper are as follows:

1) Constructed a real-time collection device for fish top and side views, achieving unconstrained measurement of fish phenotypic data in water.
2) Designed the key point skeleton structure of catfish and created the corresponding dataset.
3) Established a deep learning detection model for fish body key points and implemented the determination of 7 phenotypic data of fish bodies based on three-dimensional coordinate transformation.

The material and methods are described in Section 2. Next, a deep learning model based on Hourglass Module, the image scale parameters, and the phenotypic data acquisition is displayed in Section 3. Results and discussion are described in Section 4. Finally, Section 5 conclude the advantages and disadvantages of the proposed method in this study.

## 2. Materials and methods

### 2.1 Phenotypic data on fish

**Fig 1:**
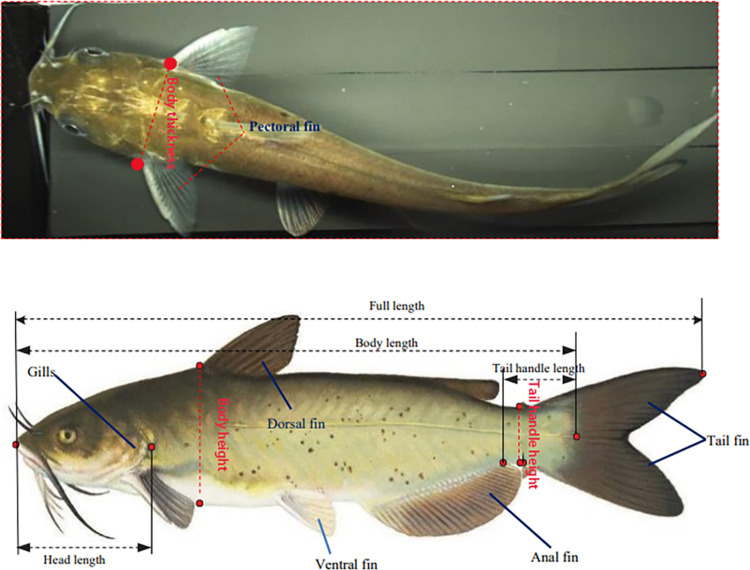
Example of phenotypic trait on channel catfish.

The research subject applied in this study is the channel catfish. The measured phenotypic trait include the body length, full length, head length, body height, tail handle length, tail handle height, and body thickness. A specific example of each type of data is shown in Fig 1.

Ethics Statement

All procedures involving live channel catfish were conducted in accordance with the ethical guidelines and approved by the Laboratory Animal Welfare and Ethics Committee of the Zhejiang Academy of Agricultural Sciences (Approval No. 25ZALAS12). During the experiments, efforts were made to minimize stress and discomfort to the fish. After measurements, the fish were returned to their original aquaculture environment without harm.

### 2.2 Hardware device structure

**Fig 2:**
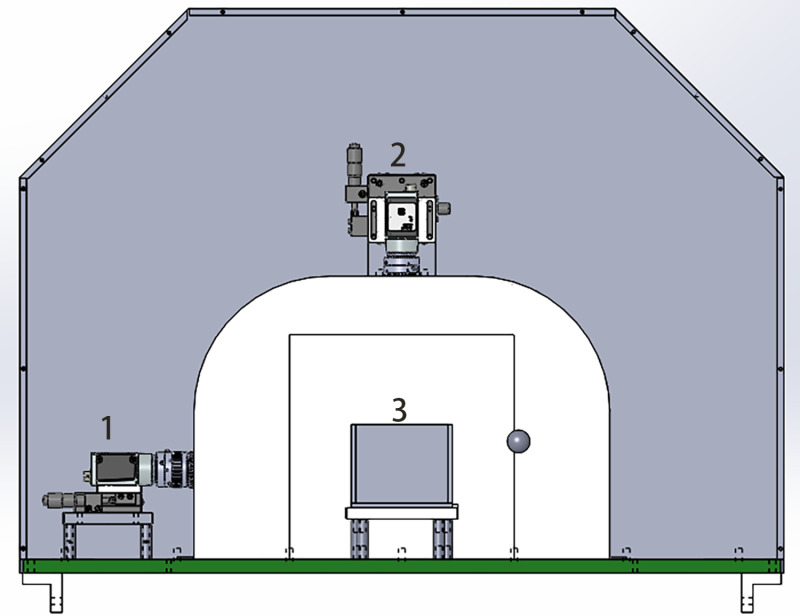
Hardware device structure. 1 is the placement location of the side-view camera; 2 is the placement location of the top-view camera; 3 is the placement location of the fish storage box.

The hardware device used in this study includes four strip light sources on the inside surface of the device, a slide track inside the device, and two four-megapixel cameras placed in a straight-up direction and toward the left direction. Fish boxes were used in this study, one side of which is transparent, and the other side is black. When measuring the phenotypic data, the fish box is filled with 3/4 volume of water before the fish is put in, and the fish box is then pushed into the device after being placed on the slide track. Based on Daheng’s industrial cameras (model: MER-503-20GC-P), we independently designed an image acquisition and testing device. The device structure is shown in Fig 2.

### 2.3 Data preparation

**Fig 3:**
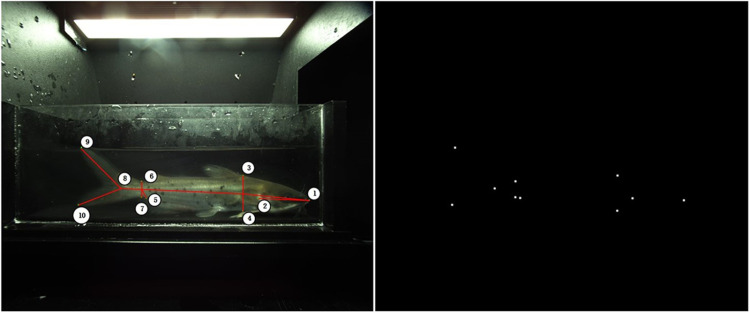
Example of a side view key point in side image.

**Fig 4:**
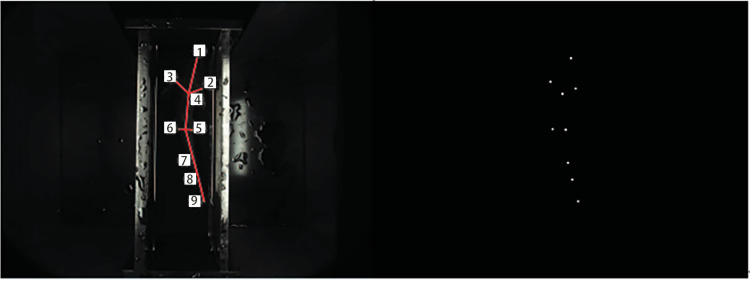
Example of a top view key point in top image.

In this study, a deep learning network based on key point positioning is adopted. To better locate the coordinates of the key points of the fish’s body, a training dataset needs to be prepared before model training. Based on different images, we defined two key point skeletons for the side and top images. To measure seven sets of phenotypic data for the fish body, we defined labels for key points. Examples of keypoints for the fish side view and top view are as follows: a total of 10 keypoints are labeled in the side view, and 9 keypoints are labeled in the top view, arranged in order according to their positions. The label descriptions are shown in Table 1, and specific examples of keypoints in the figures are shown in Figs 3 and 4.

**Table 1:** Label information of key points in top and side images.

	Label	Symbols used in the formula	Point name	Label description
Key points on side view	*1*	*S* _1_	Side-head	Fish mouth point in side image.
	*2*	*S*_2_	Head-fin	The point on the gills farthest from side-fin point.
	*3*	*S*_3_	Head-up	Inflection point between the dorsal fin and body profile.
	*4*	*S*_4_	Head-down	Corresponding point of the head-up point below the vertical line of fish body.
	*5*	*S*_5_	Front-small	Inflection point between the anal fin and body profile in side image.
	*6*	*S*_6_	Small-up	Upper point of the shortest tail handle in side image.
	*7*	*S*_7_	Small-down	Lower point of the shortest tail handle in side image.
	*8*	*S*_8_	Side-tail	Intersection point of the trace line connecting the fish body and the tail, which along the central axis of the side fish body.
	*9*	*S*_9_	Tail-up	Upper point of the tail fin.
	*10*	*S*_10_	Tail-down	Lower point of the tail fin.
Key points on top view	*1*	*T*_1_	Top-head	Fish mouth point in top image.
	*2*	*T*_2_	Fin-right	Inflection point between the right pectoral fin and body profile in top image.
	*3*	*T*_3_	Fin-left	Inflection point between the left pectoral fin and body profile in top image.
	*4*	*T*_4_	Fin-up	Corresponding point of the head-up point in top image.
	*5*	*T*_5_	Middle-right	Inflection point between the right ventral fin and body profile in top image.
	*6*	*T*_6_	Middle-left	Inflection point between the left ventral fin and body profile in top image.
	*7*	*T*_7_	Middle	Inflection point between the second dorsal fin and body profile in top image.
	*8*	*T*_8_	Small-tail	Position of shortest tail handle in top image.
	*9*	*T*_9_	Top-tail	Position of longest tail fin in top image.

### 2.4 Model inference structure

**Fig 5:**
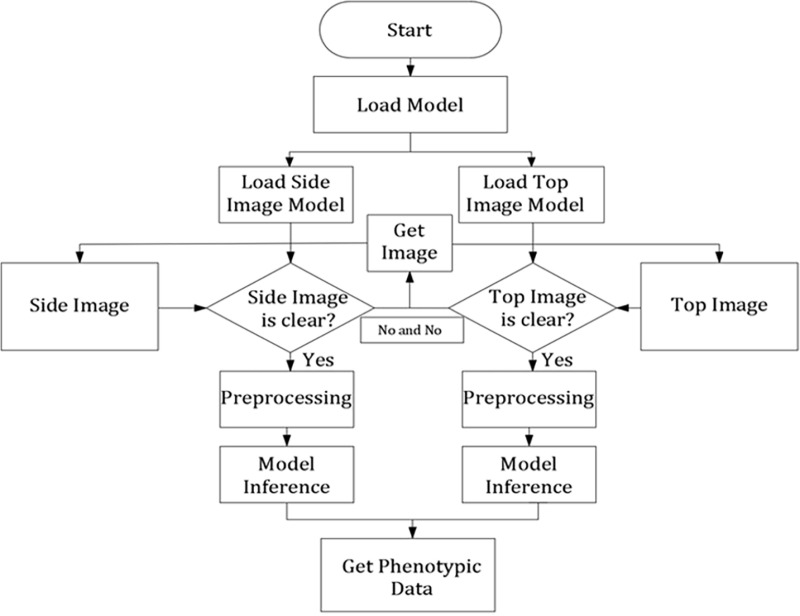
Model inference structure.

The position of channel catfish in the water does not always maintain a straight line, most of the states are curved, and the phenotypic data on the fish cannot be accurately and effectively measured using a single camera. To this end, this study is based on computer vision and combines human pose estimation methods to train key point positioning network models based on the side and top images. During the model inference, the model outputs the coordinates of the key points of individual channel catfish in the side and top images. Based on these key point coordinates, the algorithm combines the 3D coordinate transformation and scale parameters to measure the final phenotypic data. The model inference structure is shown in Fig 5.

## 3 Theory/calculation

### 3.1 Mode and training parameter

**Fig 6:**
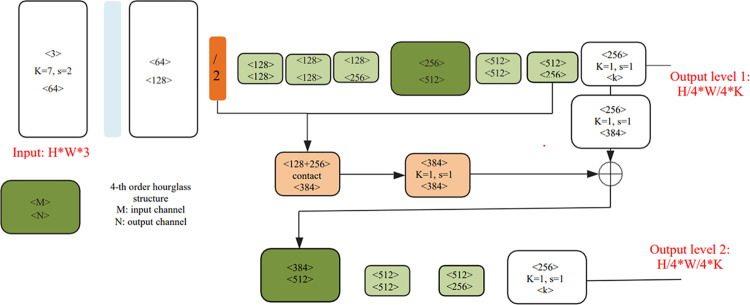
Final network structure.

**Fig 7:**
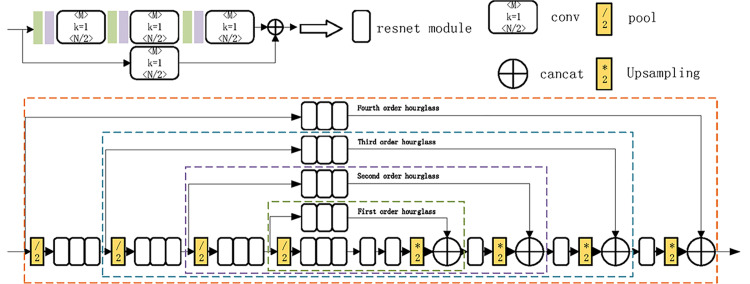
Resnet and N-th order Hourglass structure.

The structure of our network is shown in Fig 6, which consists of two fourth-order hourglass modules [30]. An Hourglass module consists of residual modules. The main design of a residual module includes shortcut connections and identity mapping; the shortcut connections make the residual possible and deepen the identity mapping of the net, allowing a gradient explosion to be avoided. The topology of an Hourglass module is symmetric, and thus for each stage presented on the way down, there is a corresponding stage going up. In the down-sampling phase, features are extracted at each scale, whereasin the up-sampling phase, the original stage features and the features obtained from up-sampling from the previous stage are passed on to the next stage, the structure of which is shown in Fig 7.

The cascaded Hourglass network module achieves accurate human body key point positioning. Compared with other methods, this deep network model based on the Hourglass network module has a concise and easy-to-expand structure, showing an accurate and efficient performance. In this study, we use two fourth-order Hourglass networks to build a key point positioning system based on the top and side views. After determining the main network model, this study trains two models to obtain key point information and prepare for the subsequent phenotypic data acquisition.

The resolution of an input image is 2,448 × 2,048 and the ground truth heatmap resolution used for training in this study is 512 × 512. The Adam optimizer is applied when optimizing the mean square error loss function, with an initial learning rate of 1e-3. During the training and inference process, we use an NVIDIA RTX2080 SUPER graphics card with a 3.70 GHz Intel i7-8700K CPU.

### 3.2 Acquisition of scale parameters

**Fig 8:**
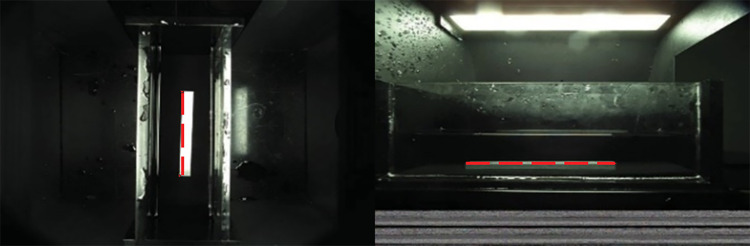
Fixed length ruler in the side and top view.


The phenotypic data on fish can be calculated based on the relation between the line segment in the image and the image scale parameters. The scale parameters between the pixel length of the ruler and the actual length are obtained by the two cameras according to the proportional relationship, an image of which is shown in Fig 8.

$$left\;scale=\frac{pixel\;length\;of\;ruler\;in\;left\;image}{truth\;length}$$ (1)

$$up\;scale=\frac{pixel\;length\;of\;ruler\;in\;top\;image}{truth\;length}$$ (2)

### 3.3 3D coordinate transformation

Because fish in water do not always maintain a straight posture, it is necessary to use 3D coordinate transformation to accurately extract their length in 3D space.

**Fig 9:**
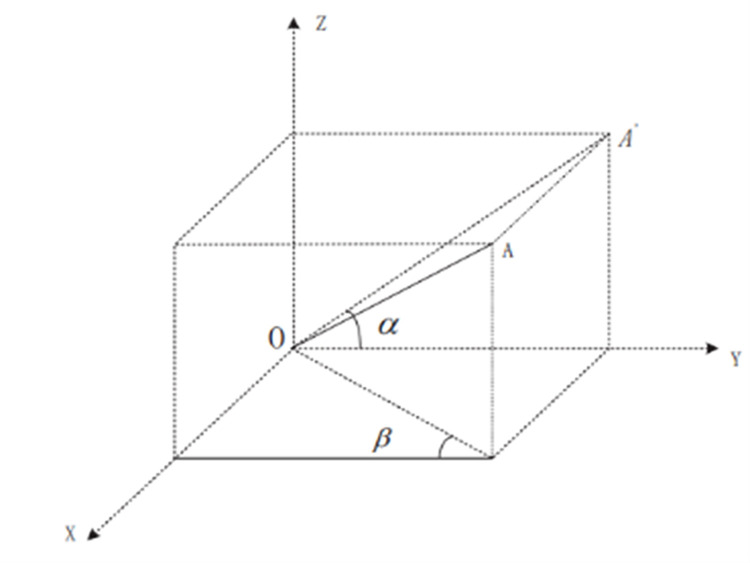
3D coordinate transformation.


In the 3D transformation shown in Fig 9, OA is the real length of the object in a 3D space, OA′ is the projection of the line segment OA on the plane YOZ, α is the angle between the line segment OA′ and the OY direction axis, and β is the angle between the projection of the line segment OA on the plane XOY and the direction axis OY. From this, the actual length of the OA can be calculated as follows:

$$\left|OA\right|=\sqrt{{\left(\frac{\left|OA^{\prime }\right|\cos\alpha }{\cos\beta }\right)}^{2}+{\left(\left|OA^{\prime }\right|*\sin\alpha \right)}^{2}}$$ (3)

### 3.4 Phenotypic data acquisition

To facilitate a better understanding of the subsequent calculation formulas, some midpoints composed of points on the side and top views, along with various functions, are added in this study. The descriptions of the points and functions are shown in Table 2.

**Table 2:** Added stuff descriptions.

Added stuff	Description
T_10_(fin-middle)	Midpoint of fin-left and fin-right line
S_11_(head-middle)	Midpoint of the head-up and head-down line
S_12_ (small-middle)	Midpoint of small-up and small-down line
T_11_(middle-middle)	Midpoint of middle-left and middle-right line
f_dist_(A, B)	Distance between two points A and B on the image
fpro(|OA’|, cosα, cosβ)	True length of the line segment OA in 3D space
Vec (A, B)	Vector from point A to point B on the image
A_Vec_(A, B)	The cosine of the angle between the vector formed by the two points A and B and the horizontal axis
VecProject(A, B)	Represents the projected length of vector A on vector B on an image
Proportion(A, B)	The proportion of the connecting line of two points A and B occupying the total length of the fin-middle to a small-tail three-section polyline in the side image

The points obtained from the side view are comprehensively processed. The side view of the fish is divided into five segments, and the cosine of the angle between each segment vector and the horizontal axis is calculated. The final phenotypic data information is calculated based on the segmentation angle and proportional relationship obtained from the top view image.

The body thickness (BT) is calculated as:


$$BT=f_{dist}(T_{2},T_{3})*up_{scale}$$ (4)

The body height (BH) is calculated as:


$$BH=f_{dist}(S_{3},S_{4})*left_{scale}$$ (5)

The tail handle height (THH) is calculated as:


$$THH=f_{dist}(S_{6},S_{7})*left_{scale}$$ (6)

The head length (HL) calculation related to a 3D coordinate transformation is as follows:


$$HL=f_{pro}(f_{dist}(S_{1},S_{2})*left_{scale},A_{Vec}(S_{1},S_{2}),A_{Vec}(T_{1},T_{10}))$$ (7)

The body length (BL) calculation is more complicated. First, the calculation involves calculating the pixel distance from the side-head point to the head-middle point.


$$lhm=f_{pro}(f_{dist}(S_{1},S_{11})*left_{scale},A_{Vec}(S_{1},S_{11}),A_{Vec}(T_{1},T_{10}))$$ (8)

Then, the calculation involves calculating the pixel distance from the head-middle point to small-middle point in the side image.


$$t_{mp}=f_{dist}((S_{11},S_{12})+f_{dist}(S_{8},S_{12}))*left_{scale}$$ (9)

Finally, the calculation combines the proportional relationship and 3D coordinate transformation to obtain the body length.


$$\begin{array}{cc} BL= & lhm+f_{pro}(t_{mp}*Proportion(T_{11},T_{10}),A_{Vec}(S_{12},S_{11}),A_{Vec}(T_{11},T_{10}))\\ & +f_{pro}(t_{mp}*Proportion(T_{7},T_{11}),A_{Vec}(S_{12},S_{11}),A_{Vec}(T_{7},T_{11}))\\ & +f_{pro}(t_{mp}*Proportion(S_{8},T_{7}),A_{Vec}(S_{12},S_{11}),A_{Vec}(T_{8},T_{7})) \end{array}$$ (10)

The calculation of the full length (FL) involves the length of the tail and the calculated body length.

The shortest distance of the projection of vectors Vec (S_9_, S_8_) and Vec (S_10_, S_8_) on vector Vec (S_12_, S_11_) is used as the tail length.


$$\begin{array}{cc} tail_{judge}= & A_{Vec}(S_{9},S_{8}),ifVecProject(Vec(S_{9},S_{8}),Vec(S_{12},S_{11}))\\ \leq & VecProject(Vec(S_{10},S_{8}),Vec(S_{12},S_{11})),else,A_{Vec}(S_{10},S_{8}) \end{array}$$ (11)

The full length (FL) is calculated as follows:


$$\begin{array}{c} FL=BL+f_{pro}(min(VecProject(Vec(S_{9},S_{8}),Vec(S_{12},S_{11})),\\ VecProject(Vec(S_{10},S_{8}),Vec(S_{12},S_{11})))*left_{scale},tail_{judge},A_{Vec}(T_{8},T_{9})) \end{array}$$ (12)

The calculation of the tail handle width (THW) involves calculating the projection of vectors Vec (S_12_, S_5_) and Vec (S_8_, S_12_) on vector Vec (S_12_, S_11_) combined with the 3D coordinate transformation to obtain the final length.


$$\begin{array}{c} THW=f_{pro}((VecProject(Vec(S_{12},S_{5}),Vec(S_{12},S_{11}))+VecProject(Vec(S_{8},S_{12}),Vec(S_{12},S_{11})))*\\ leftscale,A_{Vec}(S_{12},S_{11}),A_{Vec}(T_{8},T_{7})) \end{array}$$ (13)

### 3.5 Error evaluation

To better evaluate the accuracy of the algorithm proposed in this study for measuring of the phenotypic data of *Ictalurus punctatus*, current mainstream evaluation indicators, including the following three aspects are used.

The root mean square error (RMSE) represents the sample standard deviation among differences between estimated and measured values.


$$RMSE=\sqrt{\frac{\sum {\mathrm{\backslash nolimits}}_{i=1}^{n}|el_{i}-ml_{i\;}{|}^{2}}{n}}$$ (14)

The mean absolute error (MAE) shows how close the prediction is in the final result:


$$MAE=\frac{\sum {\mathrm{\backslash nolimits}}_{i=1}^{n}|el_{i}-ml_{i}|}{n}$$ (15)

Finally, the average relative error (MRE) indicates how large the length error is relative to the measured length:


$$MRE=\frac{\sum {\mathrm{\backslash nolimits}}_{i=1}^{n}\left(\frac{\left|el_{i}-ml_{i}\right|}{ml_{i}}\right)}{n}$$ (16)

## 4 Results and discussion

### 4.1 Positioning effect map

**Fig 10:**
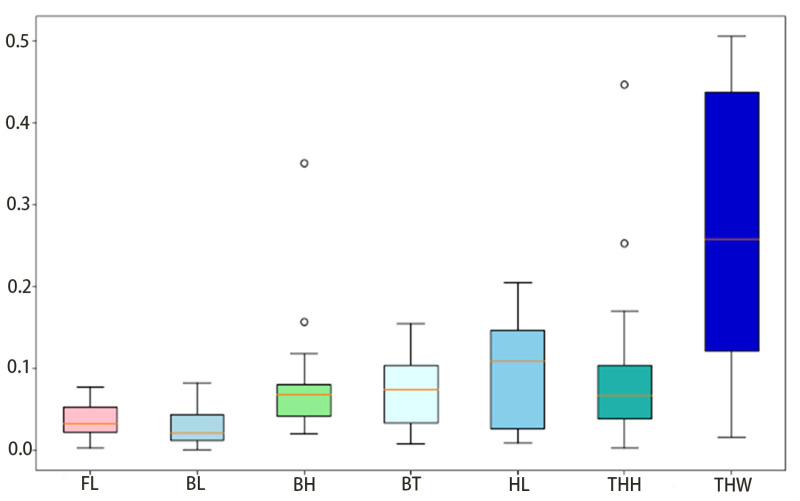
Error rate statistics.

As shown in Fig 10, the measurement method proposed in this study can effectively locate the key points of the fish’s body, even when the body is bent. This provides a reliable basis for subsequent phenotypic data calculations.

### 4.2 Overall error calculation

In this study, we measured 20 channel catfish. The true values of the phenotypic data for each channel catfish were determined by an experienced researcher. Simultaneously, the predicted values obtained through computer-based measurements were recorded in Table 3. In the table, the left seven columns represent manual measurements, while the right seven columns represent measurements obtained using the method proposed in this study.

**Table 3:** Data obtained from manual messurement vs. our proposed method.

Index	Manual measurement values							Our measurement values						
	FL (full length)	BL (body length)	BH (body height)	BT (body thickness)	HL (head length)	THH (tail handle height)	THW (tail handle width)	FL (full length)	BL (body length)	BH (body height)	BT (body thickness)	HL (head length)	THH (tail handle height)	THW (tail handle width)
1	15.97	12.919	2.429	2.12	3.406	1.735	1.102	15.258	12.914	2.378	2.448	3.653	1.564	1.281
2	14.963	11.877	2.007	2.094	3.129	1.597	1.055	14.252	11.713	2.048	2.248	3.173	1.431	0.903
3	14.988	12.122	2.398	2.135	3.126	1.726	1.1	15.434	12.696	2.144	2.343	3.46	1.833	0.607
4	14.155	11.317	2.286	2.056	2.698	1.489	1.005	14.118	11.666	2.125	2.34	3.25	1.521	1.028
5	16.342	13.457	2.6	2.352	3.211	1.952	1.183	15.916	13.399	2.378	2.429	3.364	1.934	1.245
6	14.161	11.342	2.211	2.143	2.741	1.596	0.977	13.6	12.188	2.038	2.095	3.047	1.429	1.401
7	13.569	11.09	2.189	2.033	2.704	1.515	0.961	12.521	10.808	2.038	2.084	3.06	1.131	1.08
8	14.67	11.616	3.365	2.094	2.903	1.092	1.51	14.167	11.798	2.183	2.389	3.252	1.58	1.18
9	12.949	10.234	2.028	1.847	2.517	1.504	0.89	12.174	10.064	1.868	1.832	2.443	1.248	0.617
10	12.98	10.336	2.159	1.845	2.52	1.326	0.961	12.717	10.781	2.048	2.002	2.867	1.421	0.627
11	15.248	12.069	2.478	2.274	2.902	1.748	1.069	14.857	12.452	2.374	2.503	2.865	1.655	0.949
12	13.792	11.058	2.486	2.146	2.79	1.507	1.073	13.59	11.216	2.32	2.301	3.363	1.361	1.597
13	13.071	10.216	2.184	1.858	2.518	1.581	0.897	12.961	10.94	2.041	1.932	2.891	1.523	1.276
14	15.81	12.613	2.537	2.224	2.935	1.741	1.033	16.129	12.927	2.38	2.482	3.384	1.746	1.507
15	15.466	12.471	2.43	2.231	2.748	1.69	1.115	15.828	12.559	2.38	2.454	2.71	1.926	1.097
16	16.51	13.137	2.921	2.427	3.18	2.03	1.139	16.099	13.133	2.463	2.508	3.267	1.842	1.1
17	12.971	10.334	2.22	1.894	2.38	1.424	0.962	12.163	10.4	2.133	1.989	2.431	1.368	1.143
18	12.235	9.566	1.894	1.885	2.398	1.439	0.81	11.393	9.405	2.04	2.095	2.748	1.391	1.051
19	12.012	9.544	2.072	1.824	2.453	1.067	0.811	11.408	9.131	1.828	1.764	2.432	1.113	1.216
20	10.446	8.466	1.717	1.554	2.107	1.202	0.745	11.249	9.16	1.787	1.437	2.539	1.153	1.122

The manually measured data, obtained by experienced researchers, served as the standard reference. The computer-measured data were compared against these standard values, and the measurement errors for different phenotypic data were calculated using the three error calculation methods described earlier. The results are summarized in Table 4.

**Table 4:** Error statistics table.

Category	MRE	MAE	RMSE
Full length	0.037861913	0.5167	0.579181232
Body length	0.028482984	0.304	0.388684834
Body height	0.081011836	0.20655	0.317870492
Body thickness	0.075498918	0.15595	0.180715661
Head length	0.095884419	0.25865	0.313231464
Tail handle height	0.096025598	0.14045	0.18601008
Tail handle width	0.264205244	0.25735	0.303942676
Average	0.096996	0.262807	0.324234

The mean relative error statistics are shown in Fig 10.

**Fig 11:**
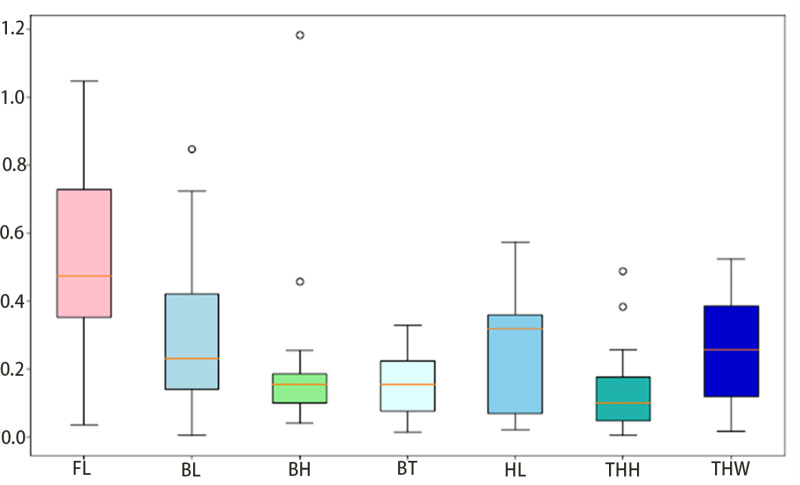
Absolute error statistics.

The mean absolute error statistics are shown in Fig 11.

In this experiment, the total length of channel catfish ranged from 12 to 17 cm. As shown in Table 4, the average relative error for total length using the proposed measurement method is approximately 3.7%, and the average absolute error is 0.5 cm, indicating high accuracy. However, a larger error was observed in the measurement of tail handle height, with a mean absolute error of 0.25 cm and a mean relative error of 26.4%, which is relatively high.

We believe that the measurement error of the tail handle height can be summarized as follows:

1) For channel catfish with a total length of 12–17 cm, the tail handle height is generally no more than 20 mm. A bias of 1 mm can result in a relative error of at least 5%.
2) The tail stem is relatively flat in some regions, making it difficult to determine precise positions, which introduces errors in manual measurements.
3) Errors were caused by a manual annotation.
4) The point coordinates, obtained from 128x128 feature maps, were upscaled by a factor of 16 to obtain the predicted coordinates in the original resolution. This caused an error of at least 0.17 cm in all phenotypic data.

Thus, errors in the scale of a few millimeters can lead to a significant average relative error.

## 5 Conclusions

In this paper, a method was proposed for measuring fish phenotypic data based on deep learning. This method applies phenotypic data measurements based on a key point detection, which uses seven phenotypic variables and achieves a high measurement accuracy. The measurement method proposed in this paper achieves an average absolute error accuracy of 0.5167 cm at full length. In addition, the body length, body height, body thickness, tail handle length, tail handle height, and head length were measured with relatively good accuracy. However, a large error may occur in measuring the tail handle length. We will explore more effective ways to reduce this error in future studies. It is worth noting that although the research subject applied in this study is channel catfish, the measurement method proposed herein can be applied to the measurement of phenotypic data of other types of fish.

Novelties of the study: 1) In-water measurement. Traditional methods for measuring fish phenotype data are typically conducted out of water. This study developed a hardware measurement device capable of measuring fish phenotype data in water, significantly reducing the stress response of fish and improving animal welfare; 2) Dataset construction. Based on the phenotype data of the target fish, this paper designed the skeletal structures for the side and top views of the fish body. Key points on the fish body were annotated, and a comprehensive image dataset was constructed to support deep learning model training; 3) Comprehensive phenotype measurement. Traditional methods are often limited to measuring body length and width. This study expands the scope by simultaneously measuring seven sets of phenotypic data, providing a more comprehensive characterization of fish morphology.

Shortcomings of the method: 1) Measurement errors. Experimental data indicate that the relative errors for tail stem width and thickness measurements are relatively large. As discussed in Section 4.2, the width and thickness of the tail are usually measured in a deformed state; 2) Hardware Limitations. The resolution of the industrial cameras used for capturing fish images may limit measurement accuracy. While increasing camera resolution could improve results, it would also raise equipment costs. A balance between cost and measurement accuracy must be carefully considered to meet application requirements. Based on the constructed fish phenotype measurement device, the experimental computer can determine 7 sets of fish phenotype data within 1 second, meeting the demand for a large amount of phenotype data in aquatic animal breeding and optimization, which is beneficial for fisheries experts to combine with genes to cultivate new varieties. Although the research object of this experiment is catfish, the device can be adapted in the future by adjusting the detection model for key points on fish bodies. This adjustment would enable the determination of phenotype data for other aquatic animals, thereby meeting the needs of breeding professionals in a wider range of fields.

## Supporting information

S1_Fig Top-view and side-view photographs of 20 channel catfish. (ZIP)
