# Supplementary figures and images for: Point-based method for measuring the phenotypic data of channel catfish (Ictalurus punctatus)
Source: PLoS One. 2025 Jun 5;20(6):e0324158. doi: 10.1371/journal.pone.0324158 (PMC12140260; doi:10.1371/journal.pone.0324158)

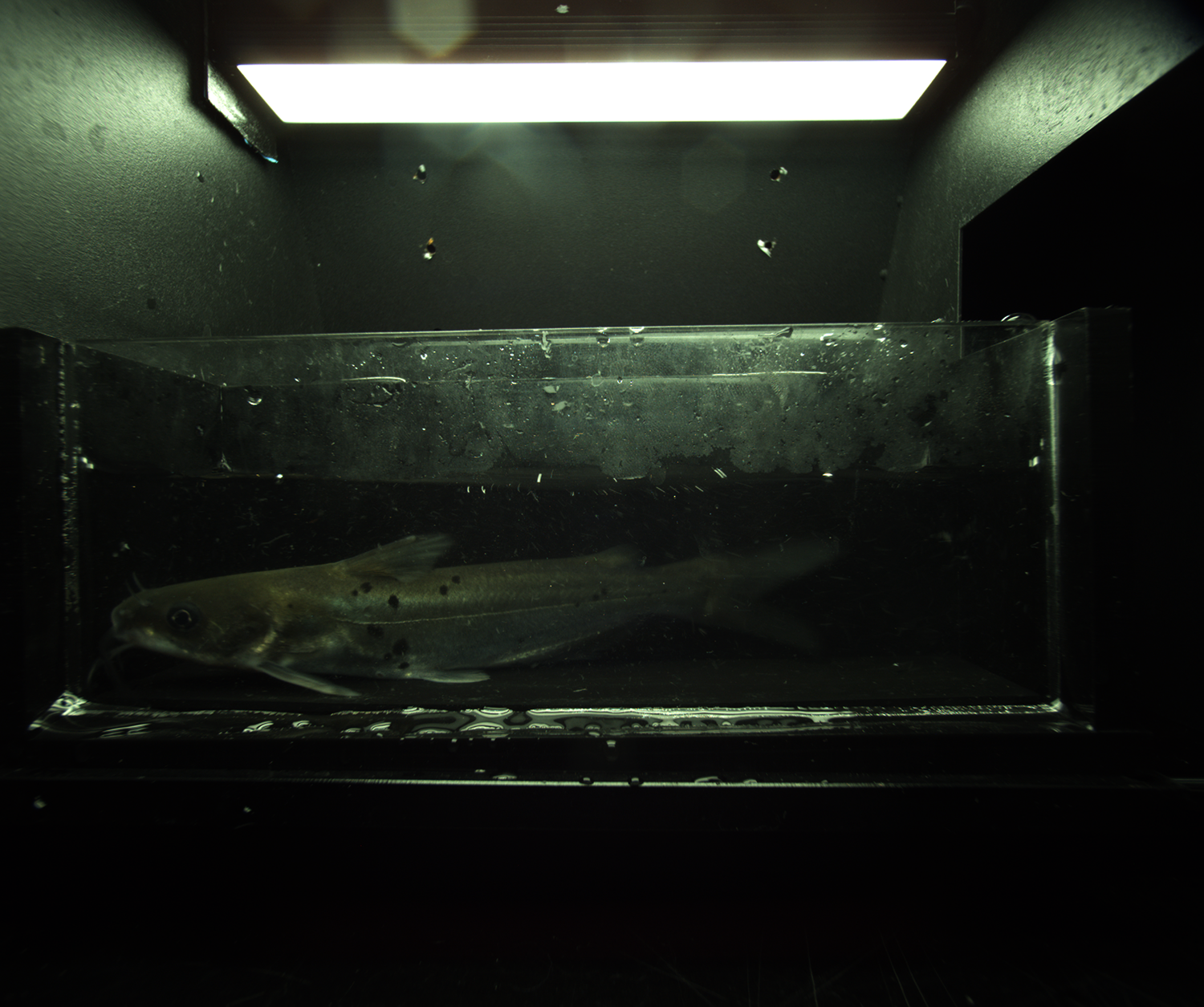

Supplement: S1_Fig — (ZIP) [file pone.0324158.s001.zip › S1_Fig/side view/12.tif]

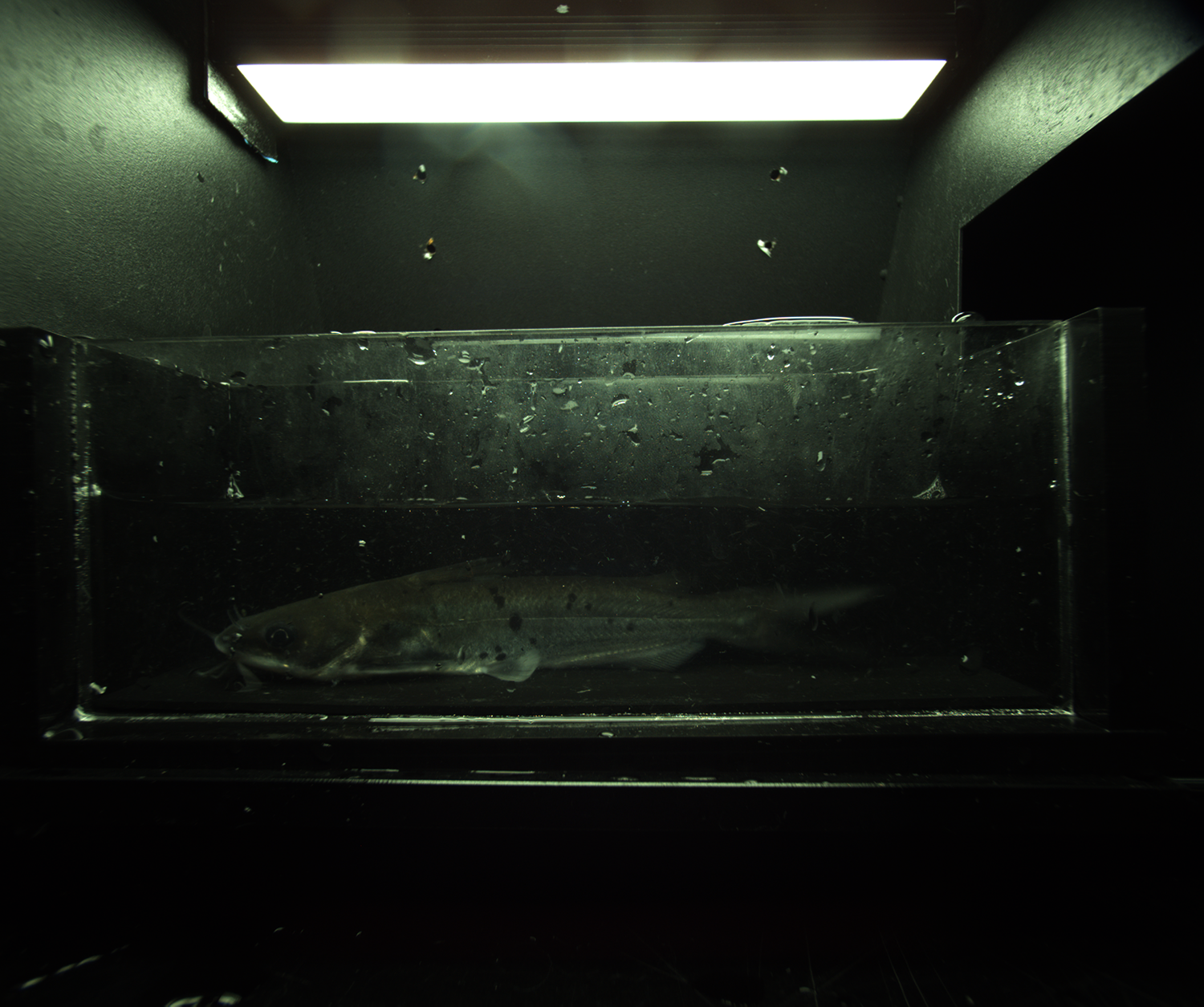

Supplement: S1_Fig — (ZIP) [file pone.0324158.s001.zip › S1_Fig/side view/13.tif]

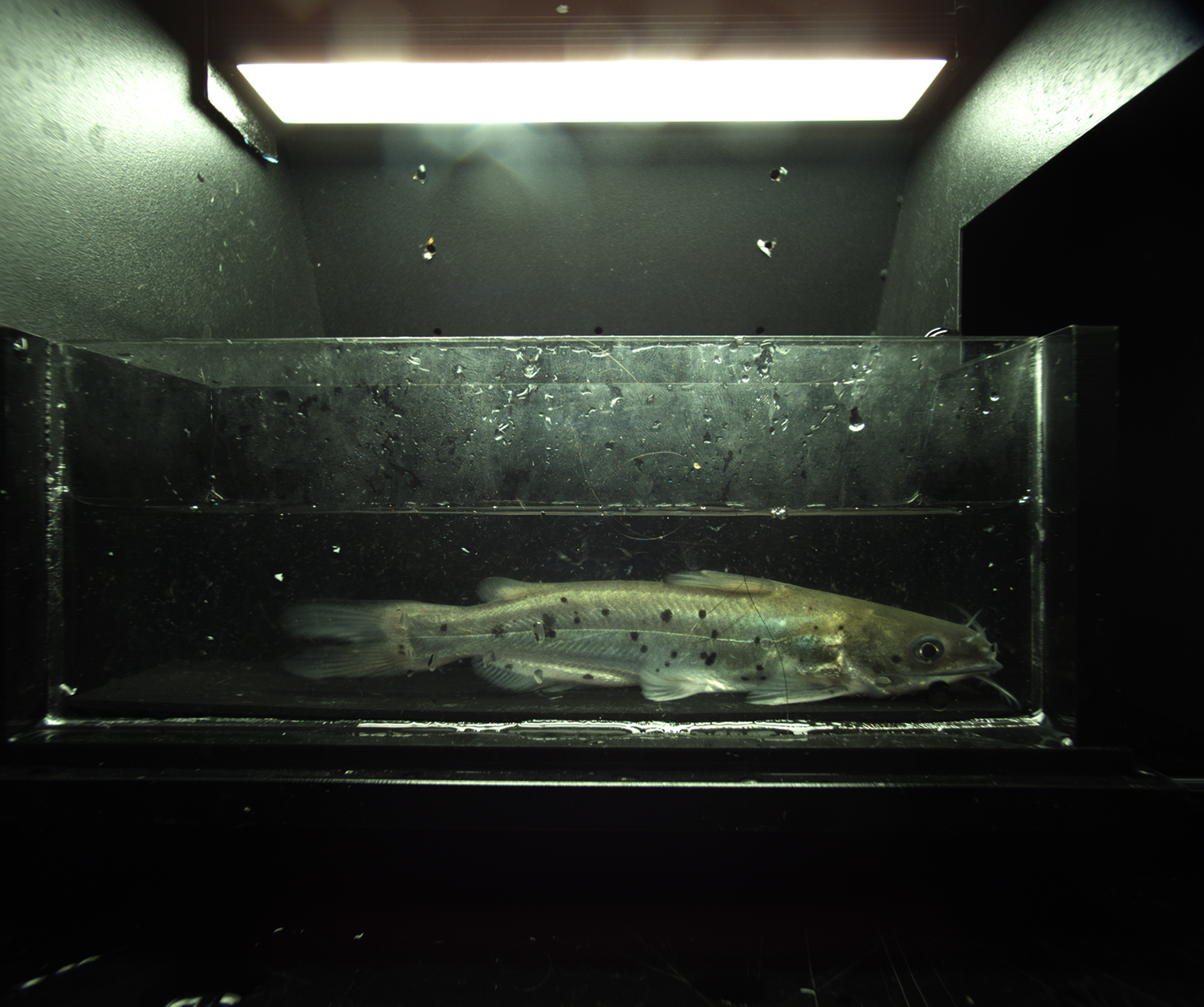

Supplement: S1_Fig — (ZIP) [file pone.0324158.s001.zip › S1_Fig/side view/14.tif]

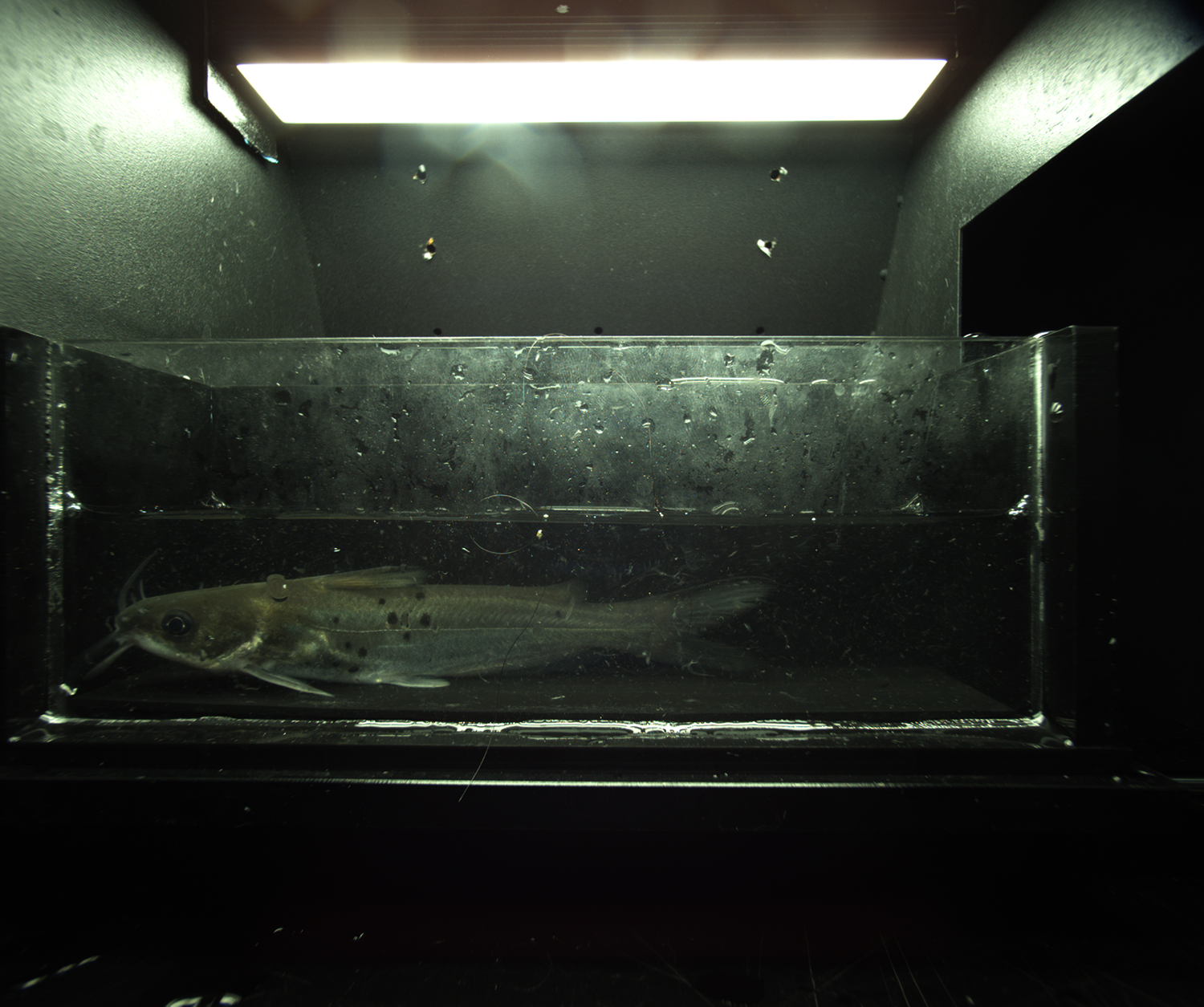

Supplement: S1_Fig — (ZIP) [file pone.0324158.s001.zip › S1_Fig/side view/15.tif]

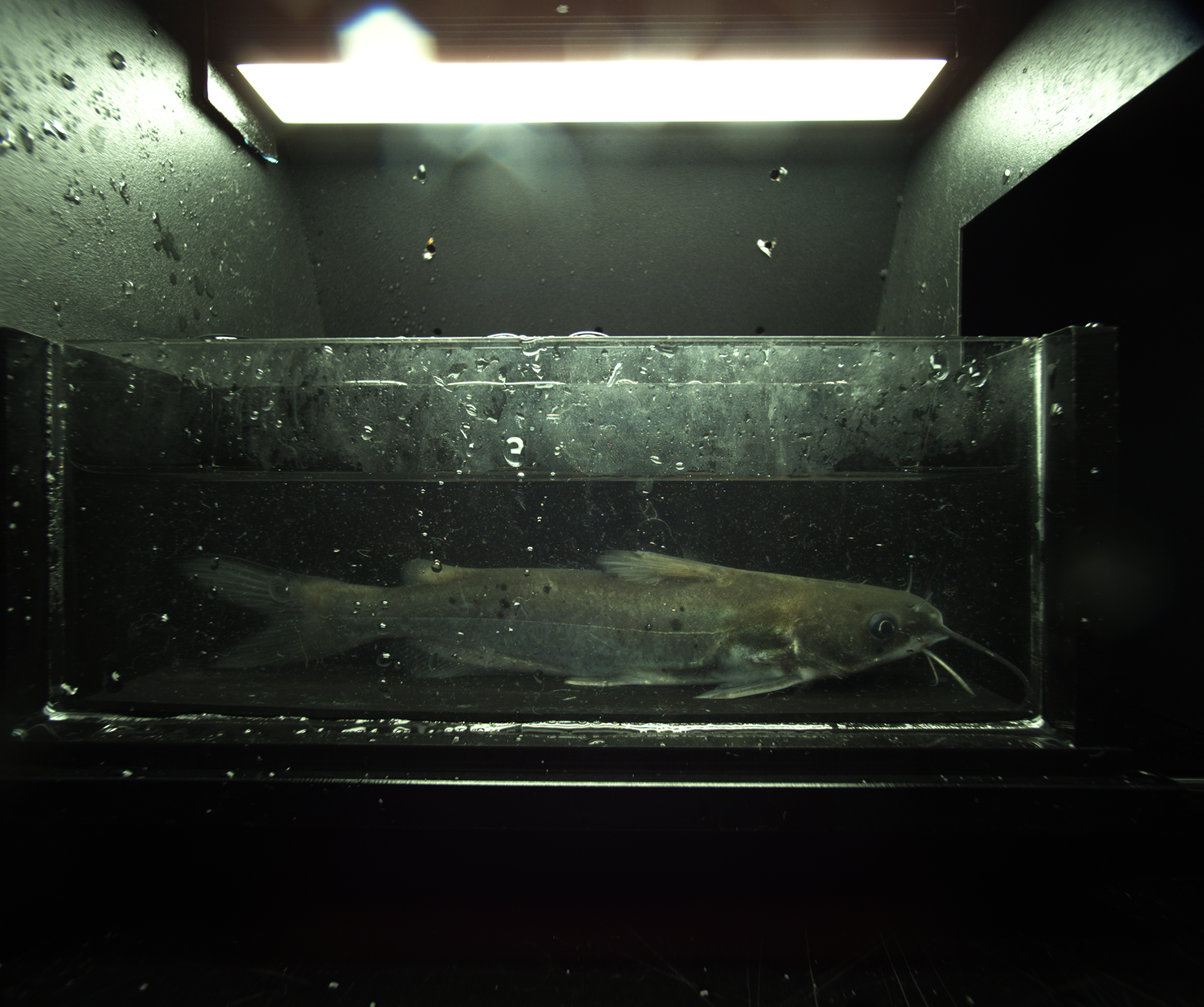

Supplement: S1_Fig — (ZIP) [file pone.0324158.s001.zip › S1_Fig/side view/16.tif]

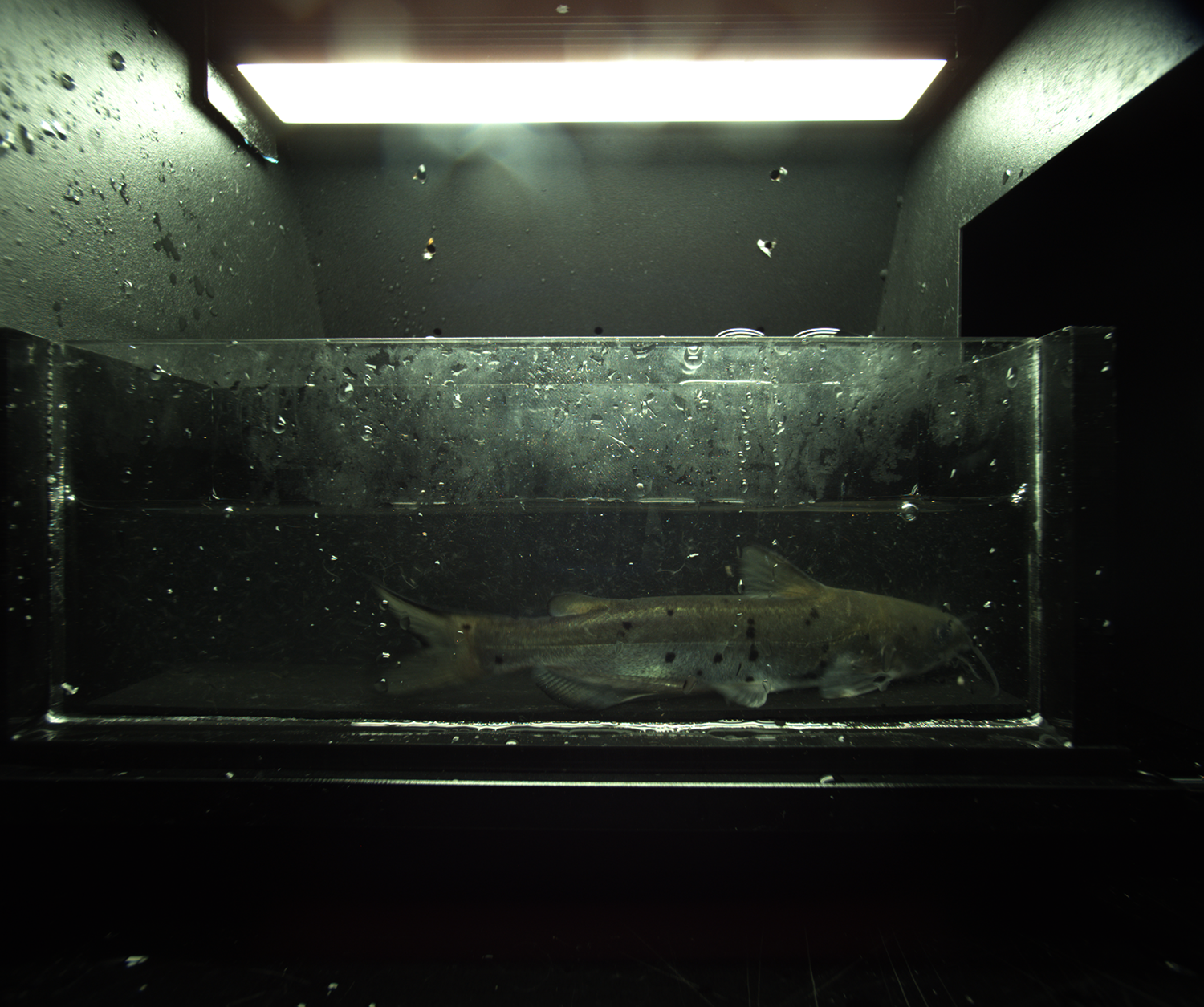

Supplement: S1_Fig — (ZIP) [file pone.0324158.s001.zip › S1_Fig/side view/17.tif]

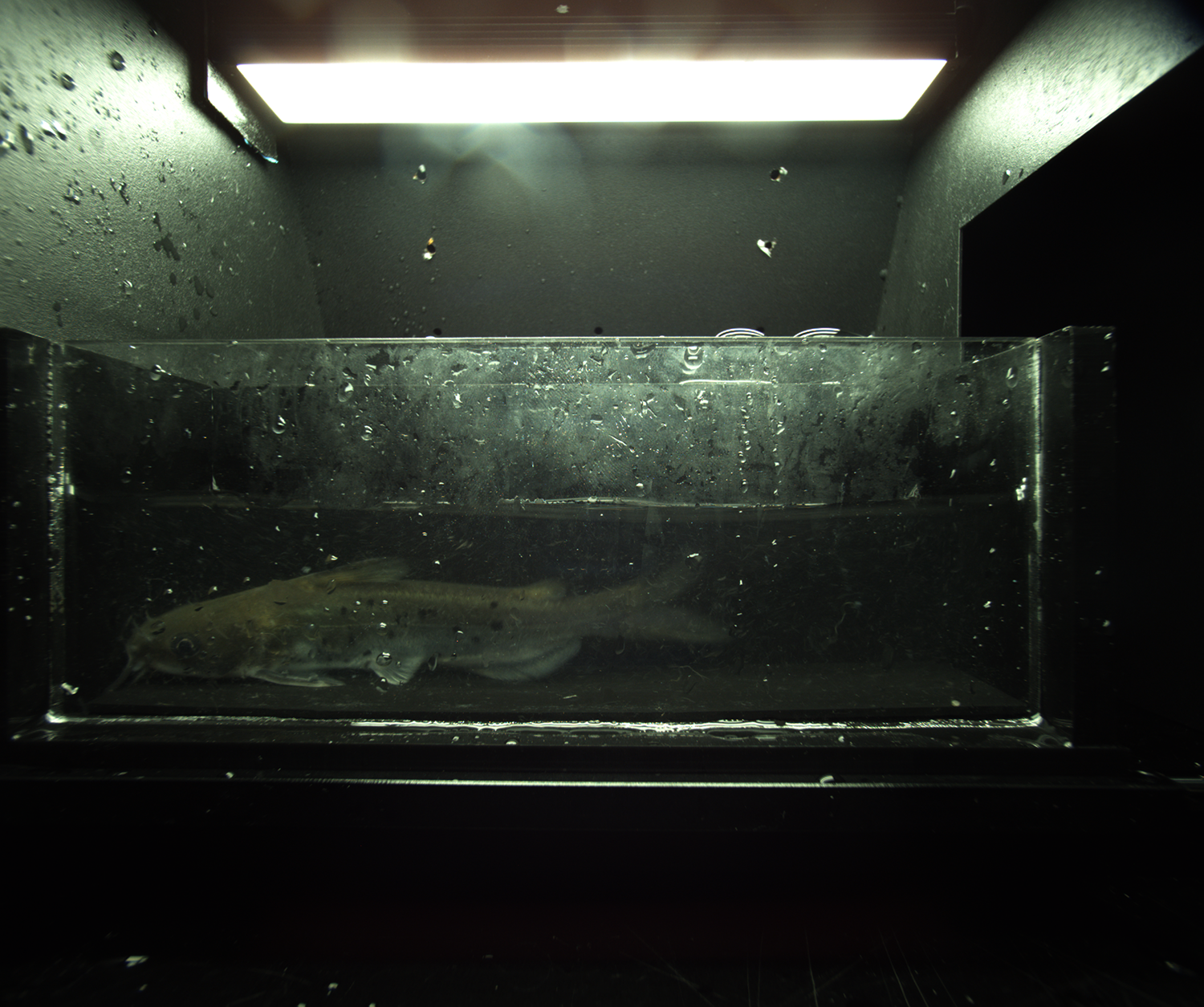

Supplement: S1_Fig — (ZIP) [file pone.0324158.s001.zip › S1_Fig/side view/18.tif]

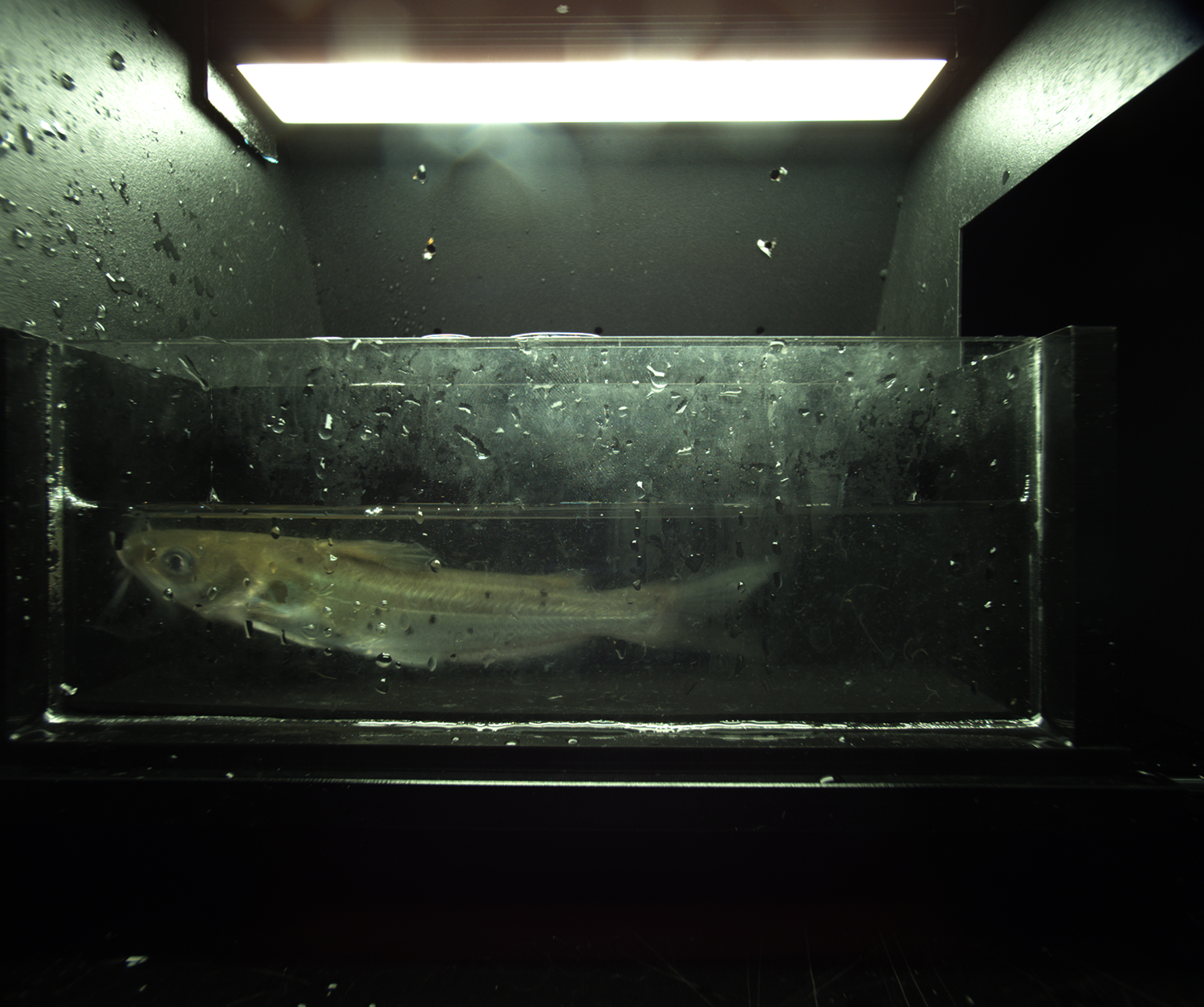

Supplement: S1_Fig — (ZIP) [file pone.0324158.s001.zip › S1_Fig/side view/19.tif]

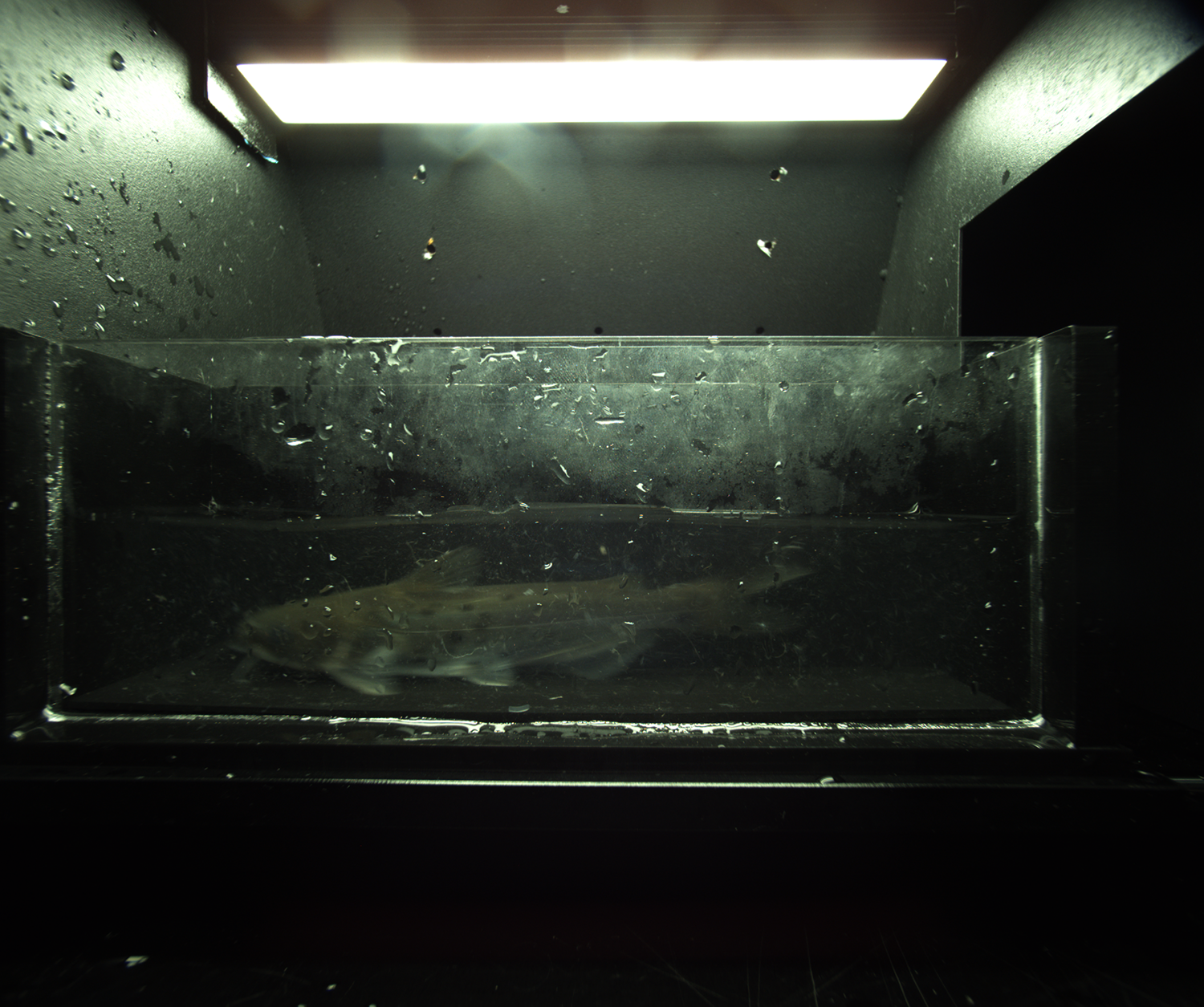

Supplement: S1_Fig — (ZIP) [file pone.0324158.s001.zip › S1_Fig/side view/20.tif]

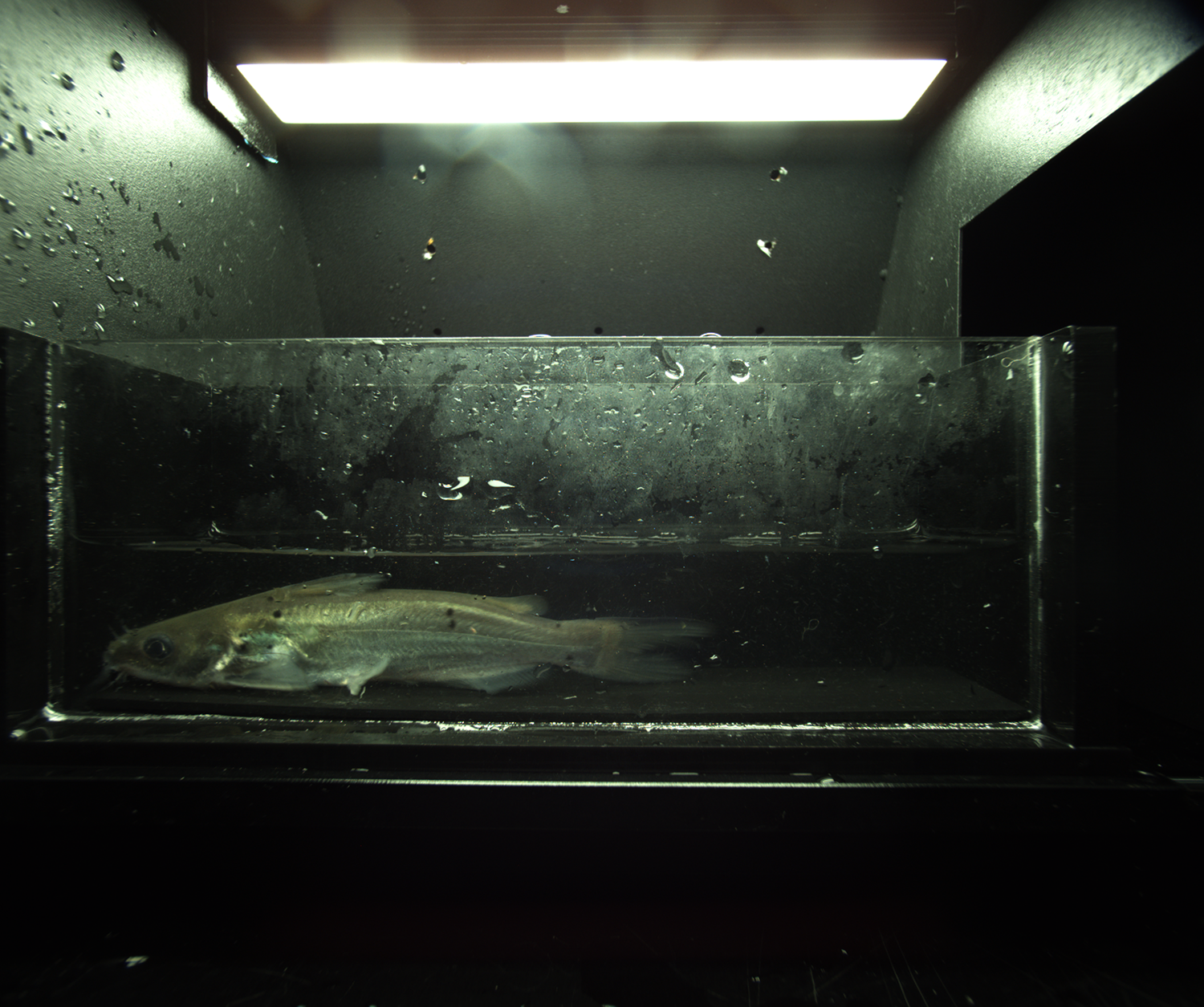

Supplement: S1_Fig — (ZIP) [file pone.0324158.s001.zip › S1_Fig/side view/21.tif]

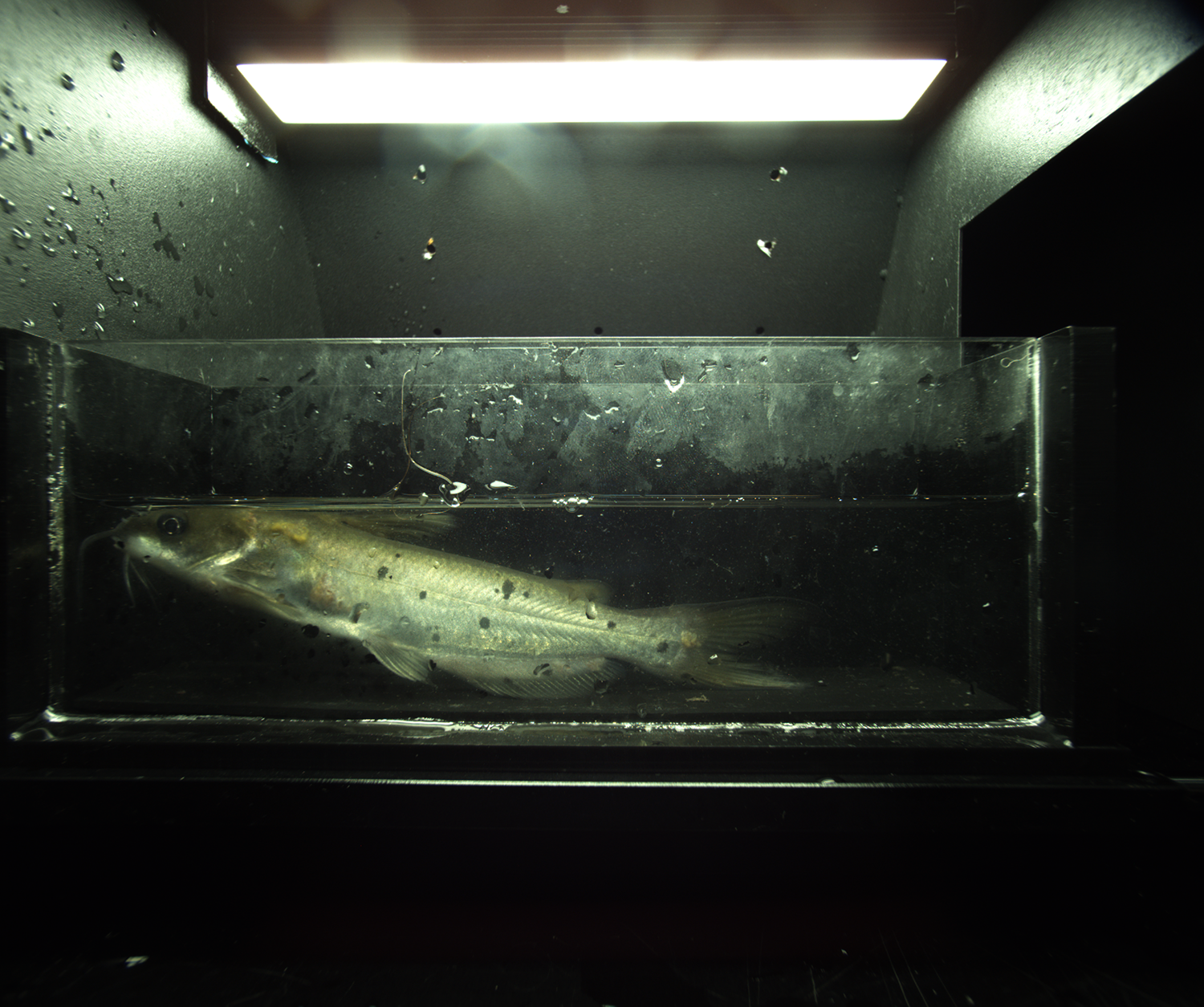

Supplement: S1_Fig — (ZIP) [file pone.0324158.s001.zip › S1_Fig/side view/22.tif]

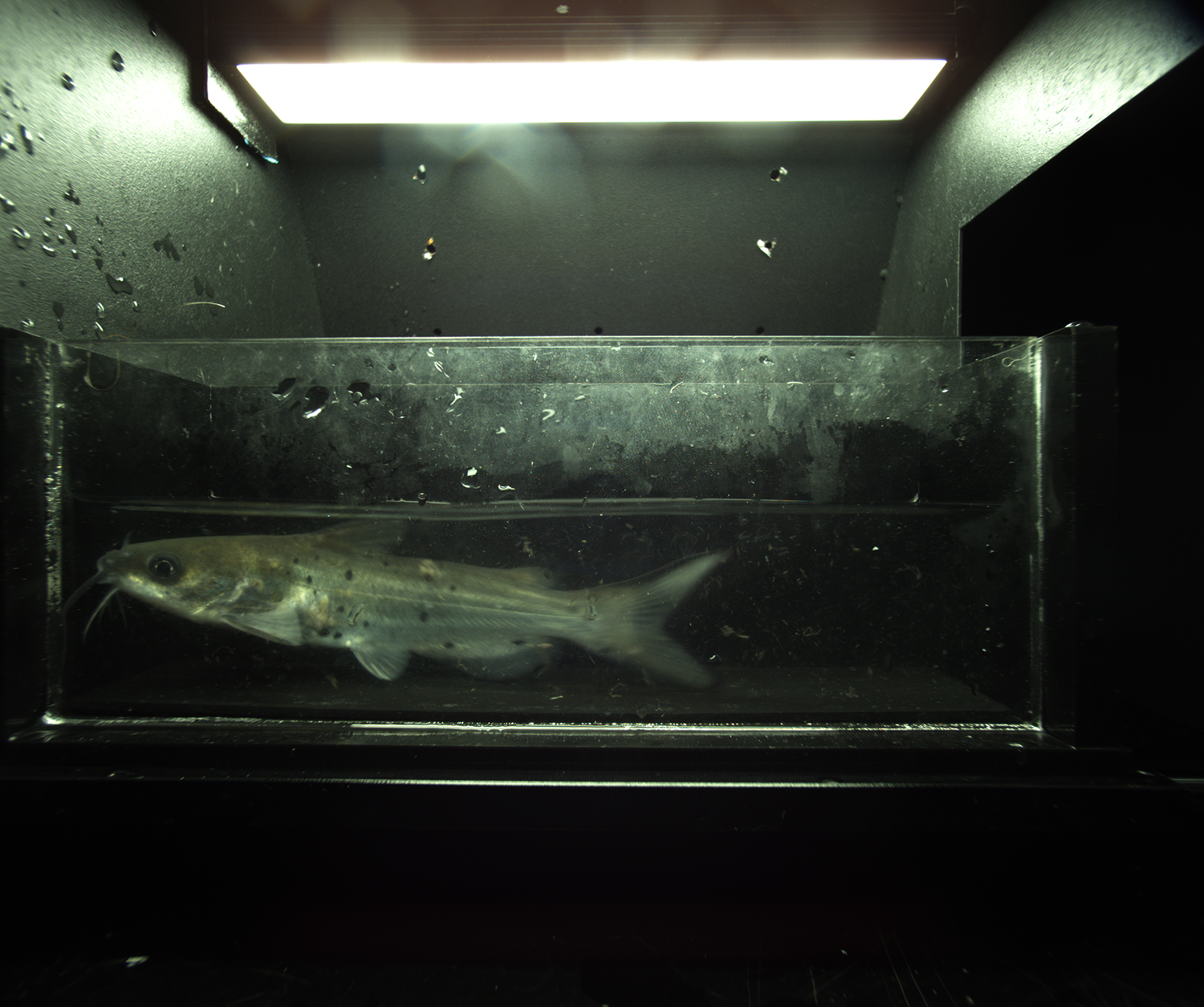

Supplement: S1_Fig — (ZIP) [file pone.0324158.s001.zip › S1_Fig/side view/23.tif]

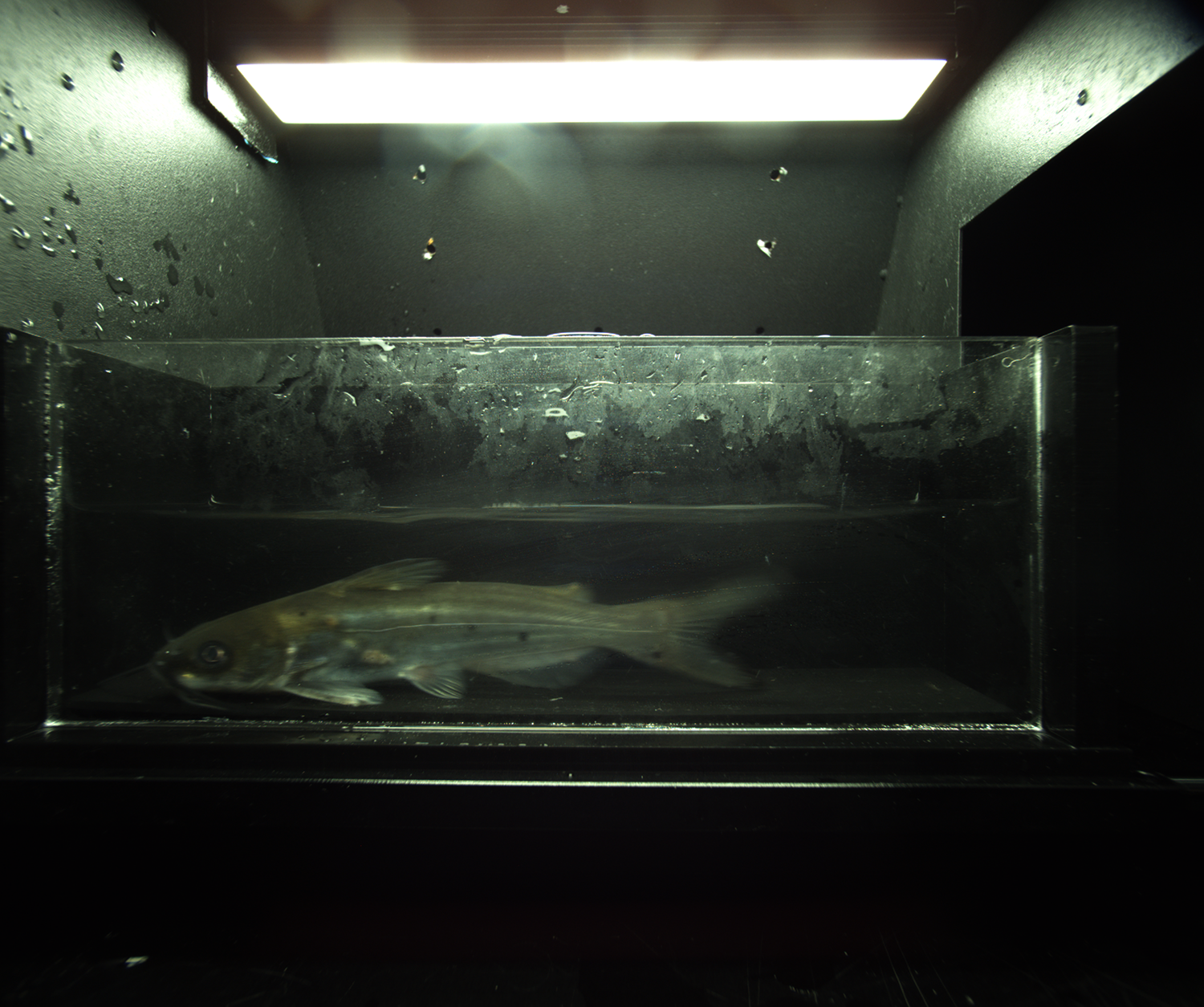

Supplement: S1_Fig — (ZIP) [file pone.0324158.s001.zip › S1_Fig/side view/24.tif]

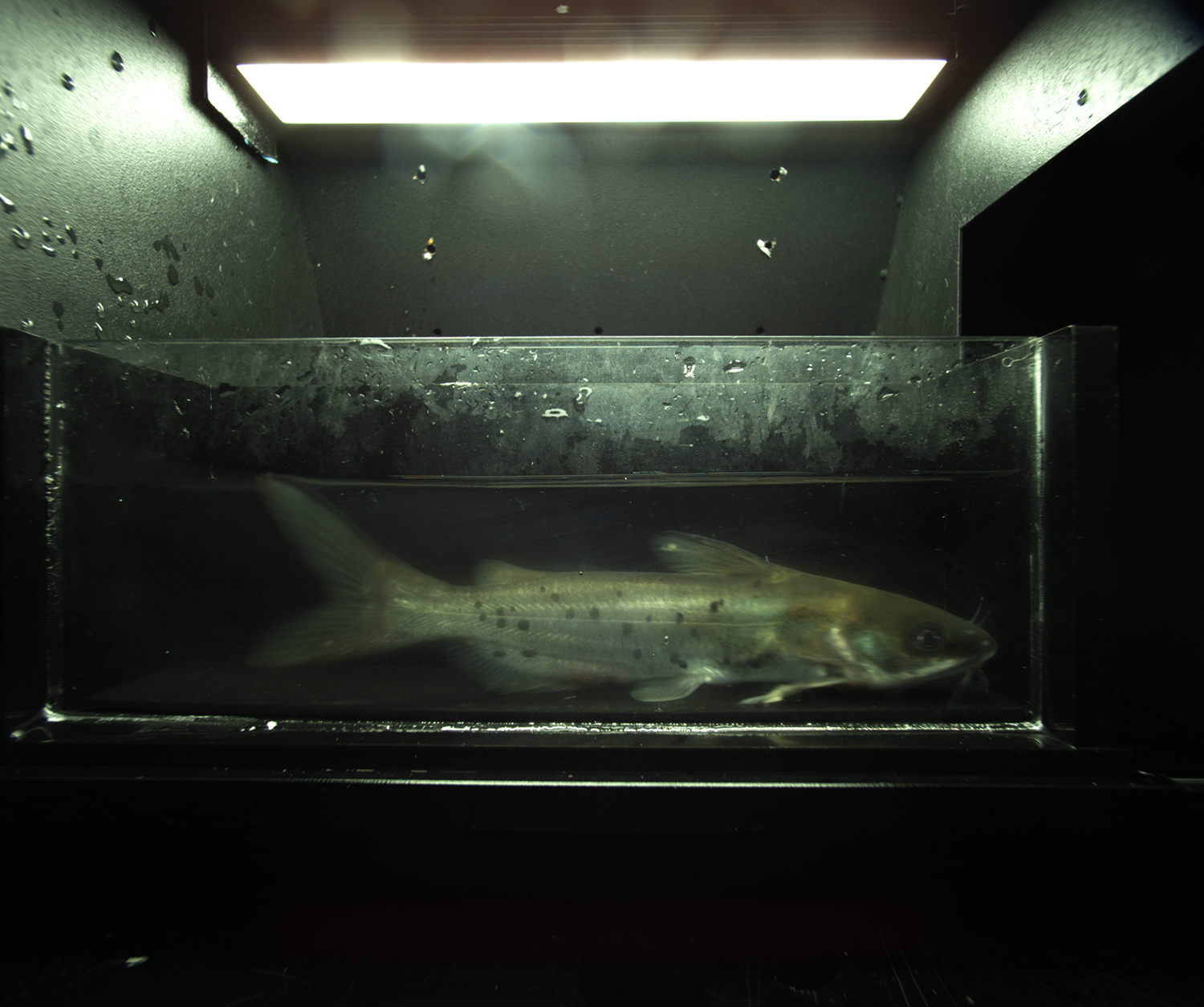

Supplement: S1_Fig — (ZIP) [file pone.0324158.s001.zip › S1_Fig/side view/25.tif]

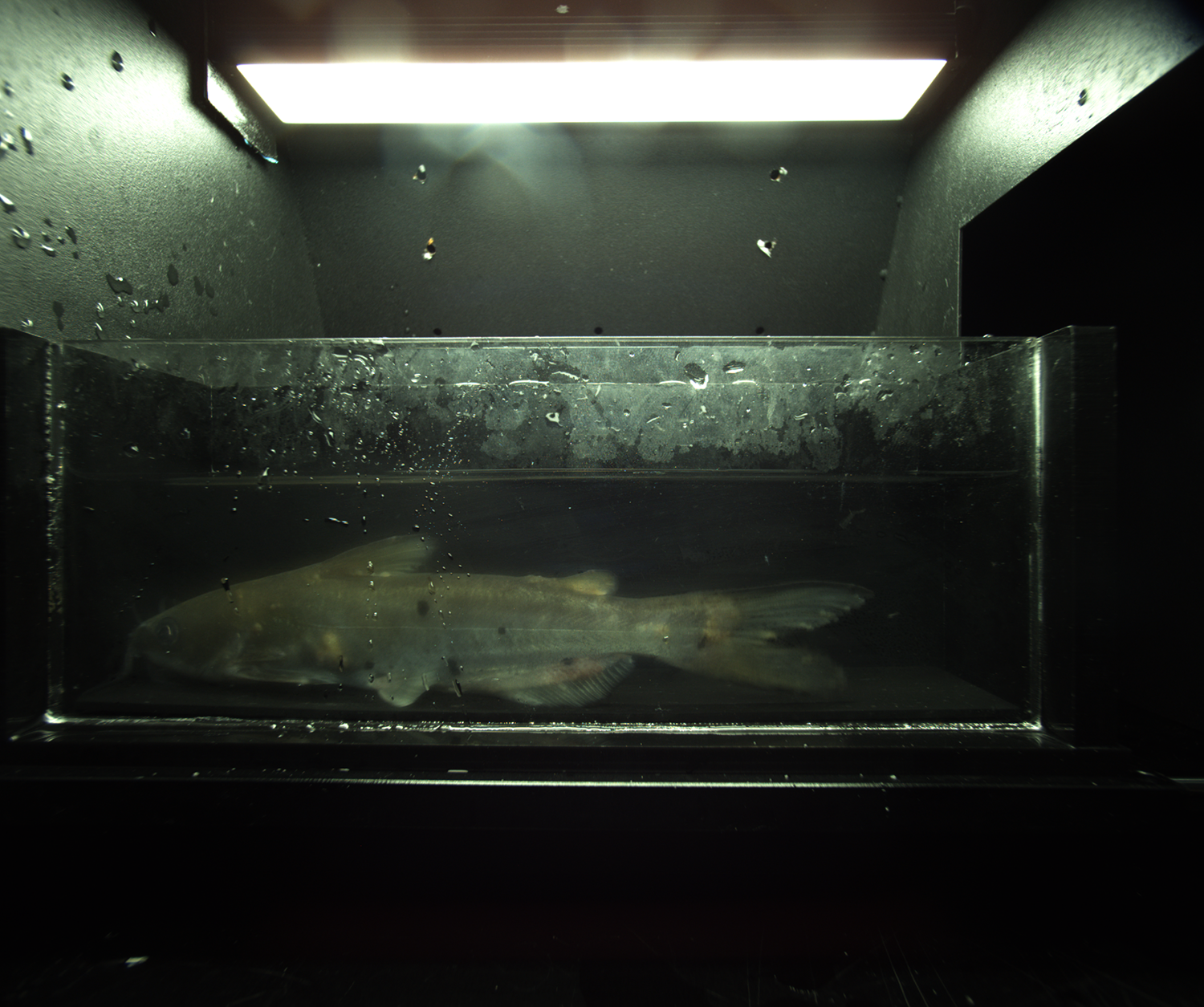

Supplement: S1_Fig — (ZIP) [file pone.0324158.s001.zip › S1_Fig/side view/26.tif]

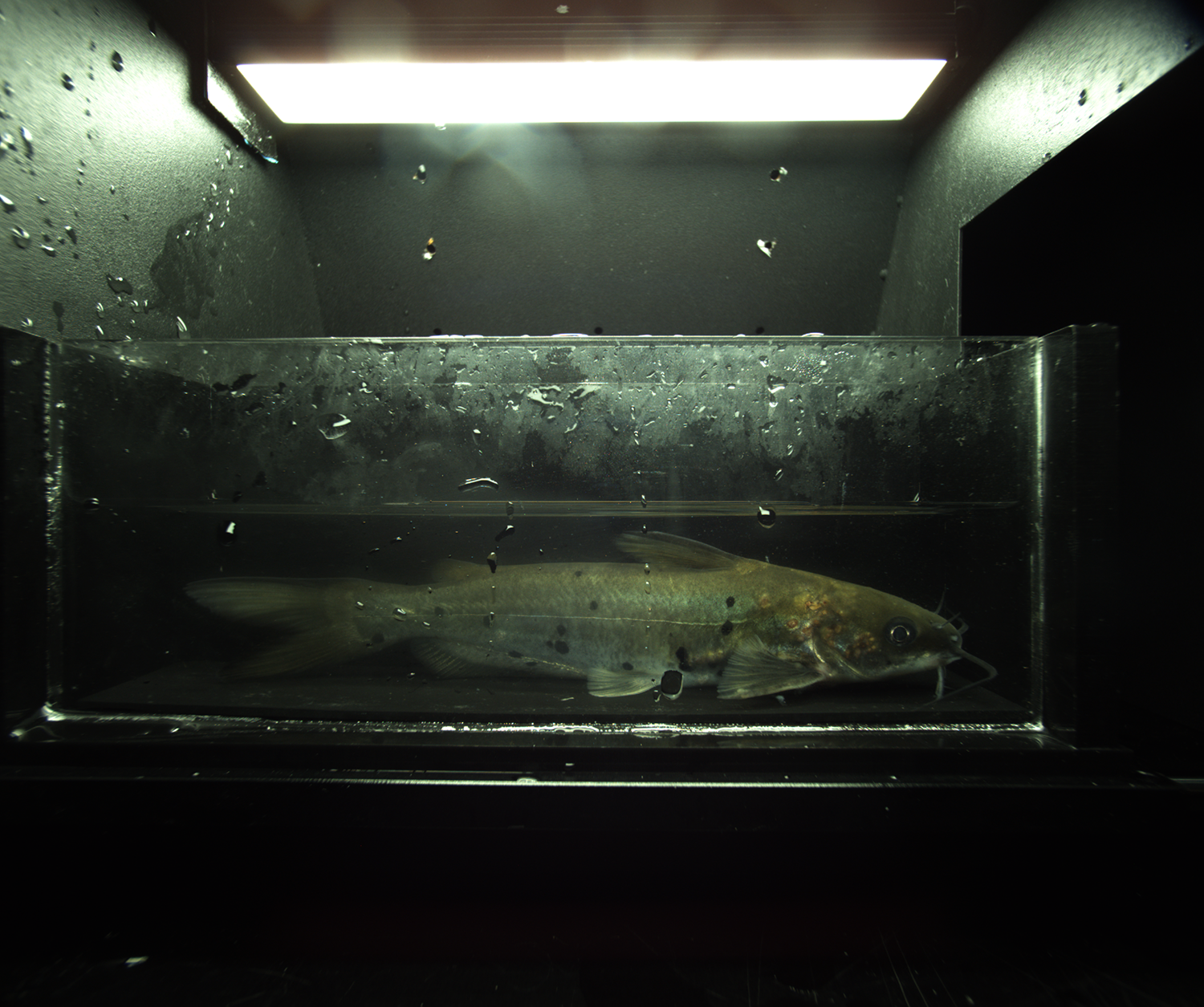

Supplement: S1_Fig — (ZIP) [file pone.0324158.s001.zip › S1_Fig/side view/27.tif]

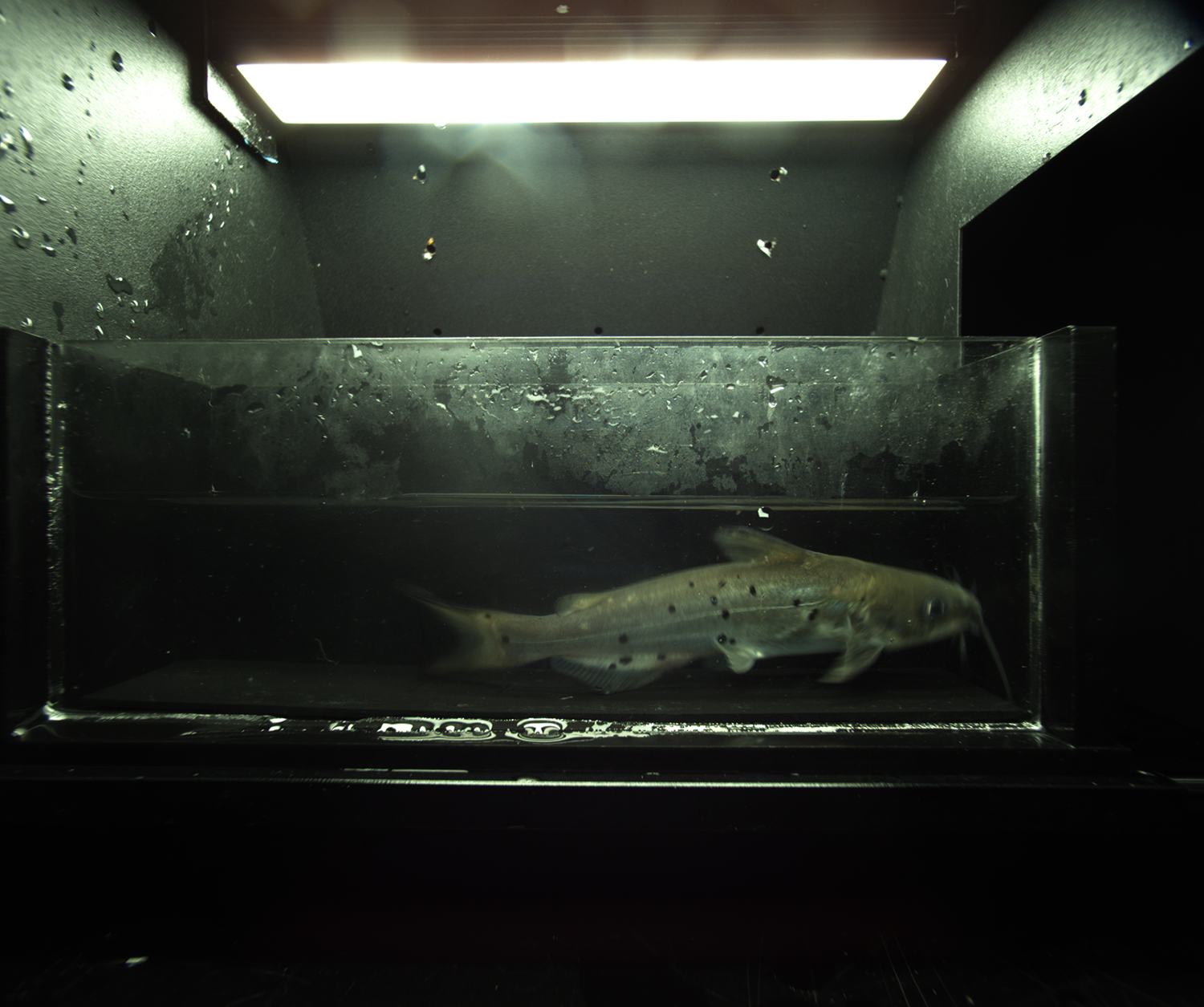

Supplement: S1_Fig — (ZIP) [file pone.0324158.s001.zip › S1_Fig/side view/28.tif]

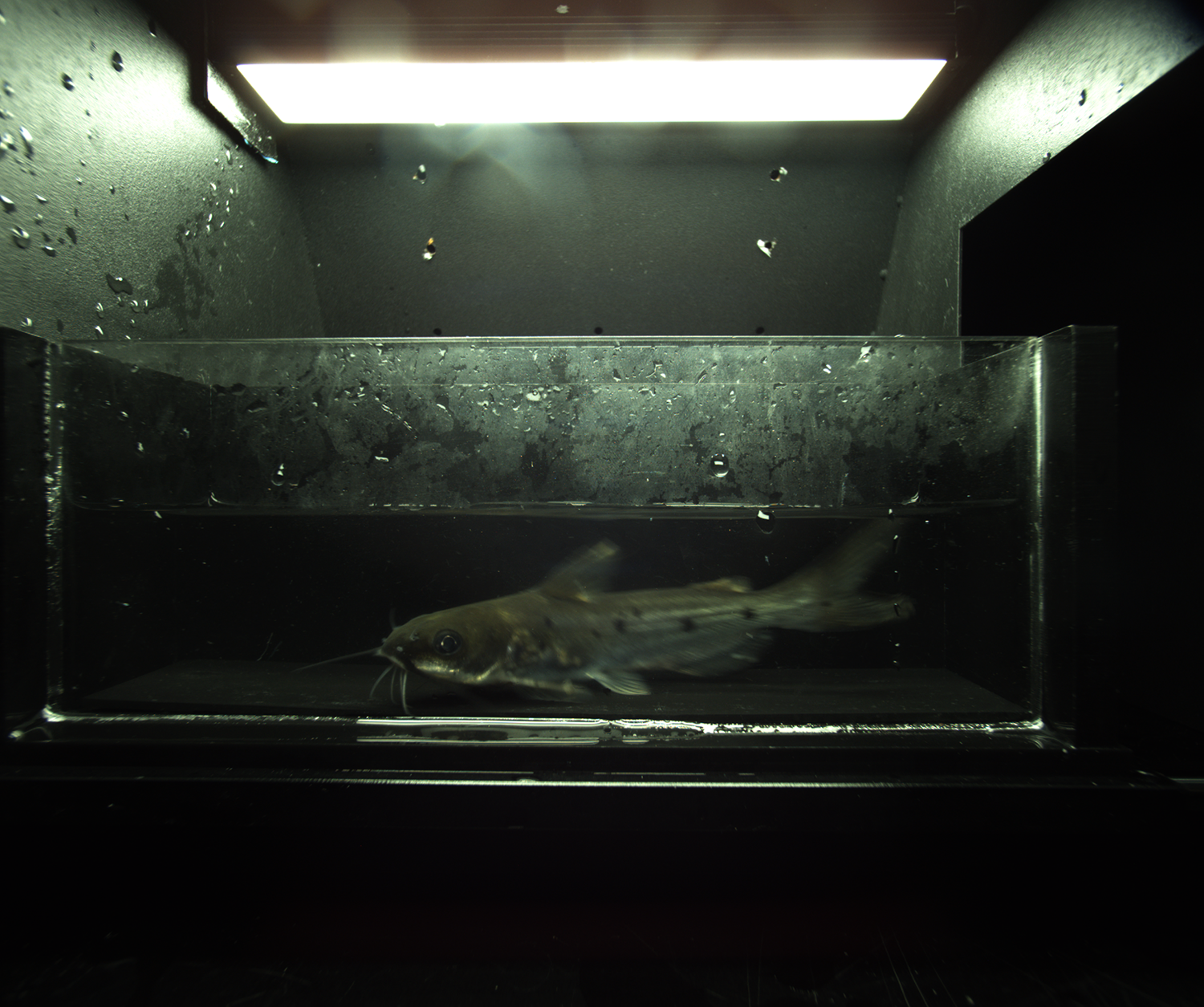

Supplement: S1_Fig — (ZIP) [file pone.0324158.s001.zip › S1_Fig/side view/29.tif]

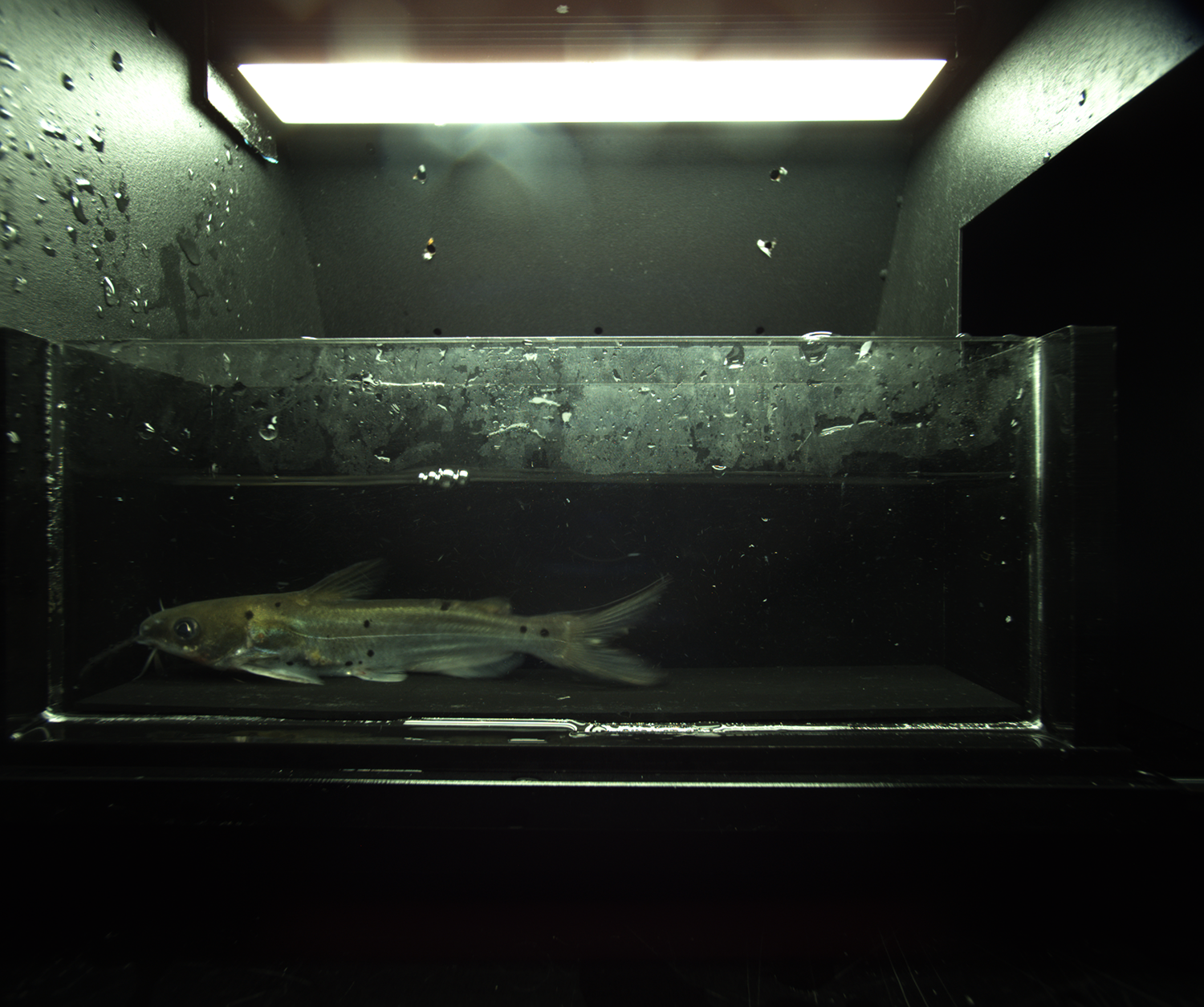

Supplement: S1_Fig — (ZIP) [file pone.0324158.s001.zip › S1_Fig/side view/30.tif]

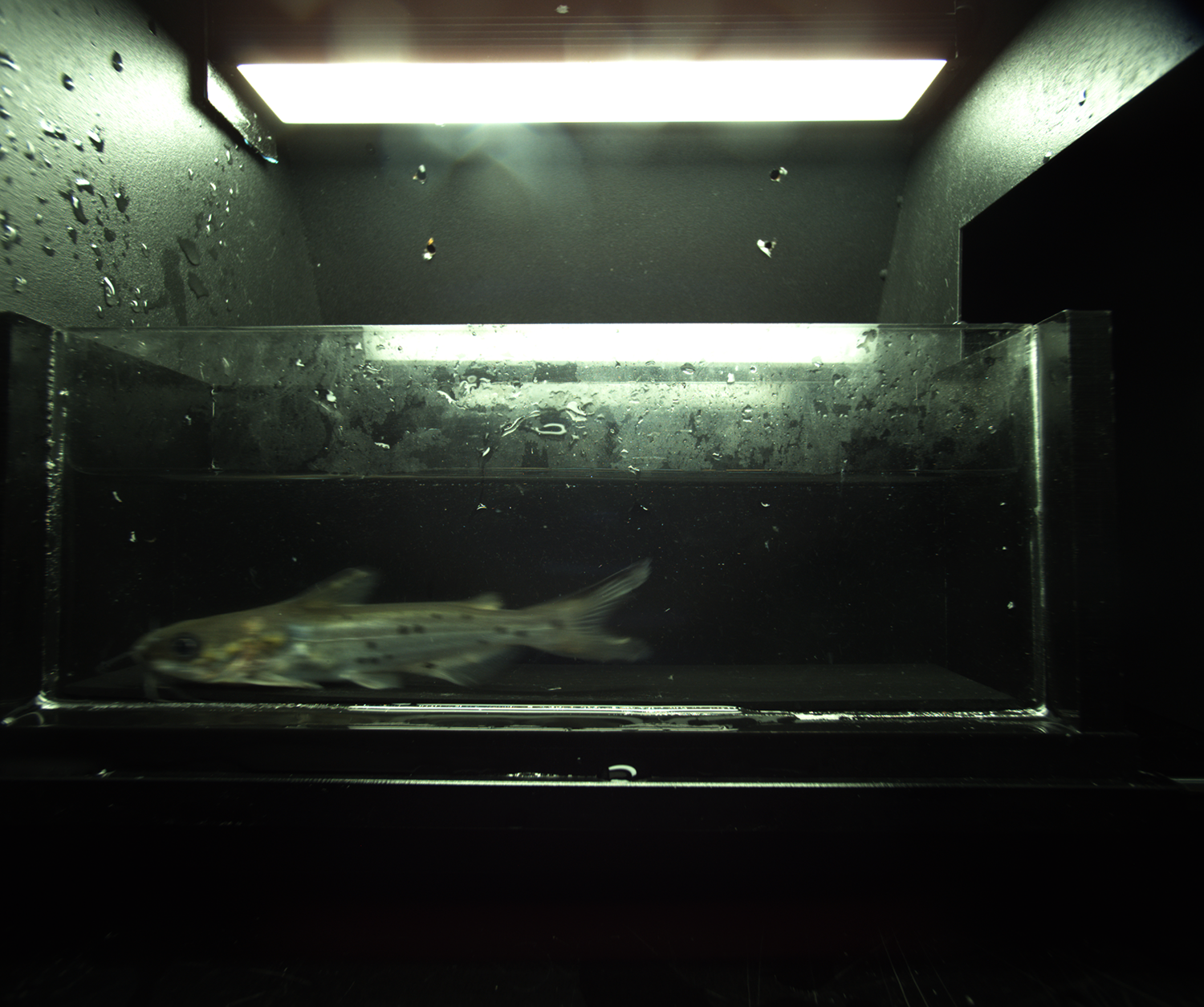

Supplement: S1_Fig — (ZIP) [file pone.0324158.s001.zip › S1_Fig/side view/31.tif]

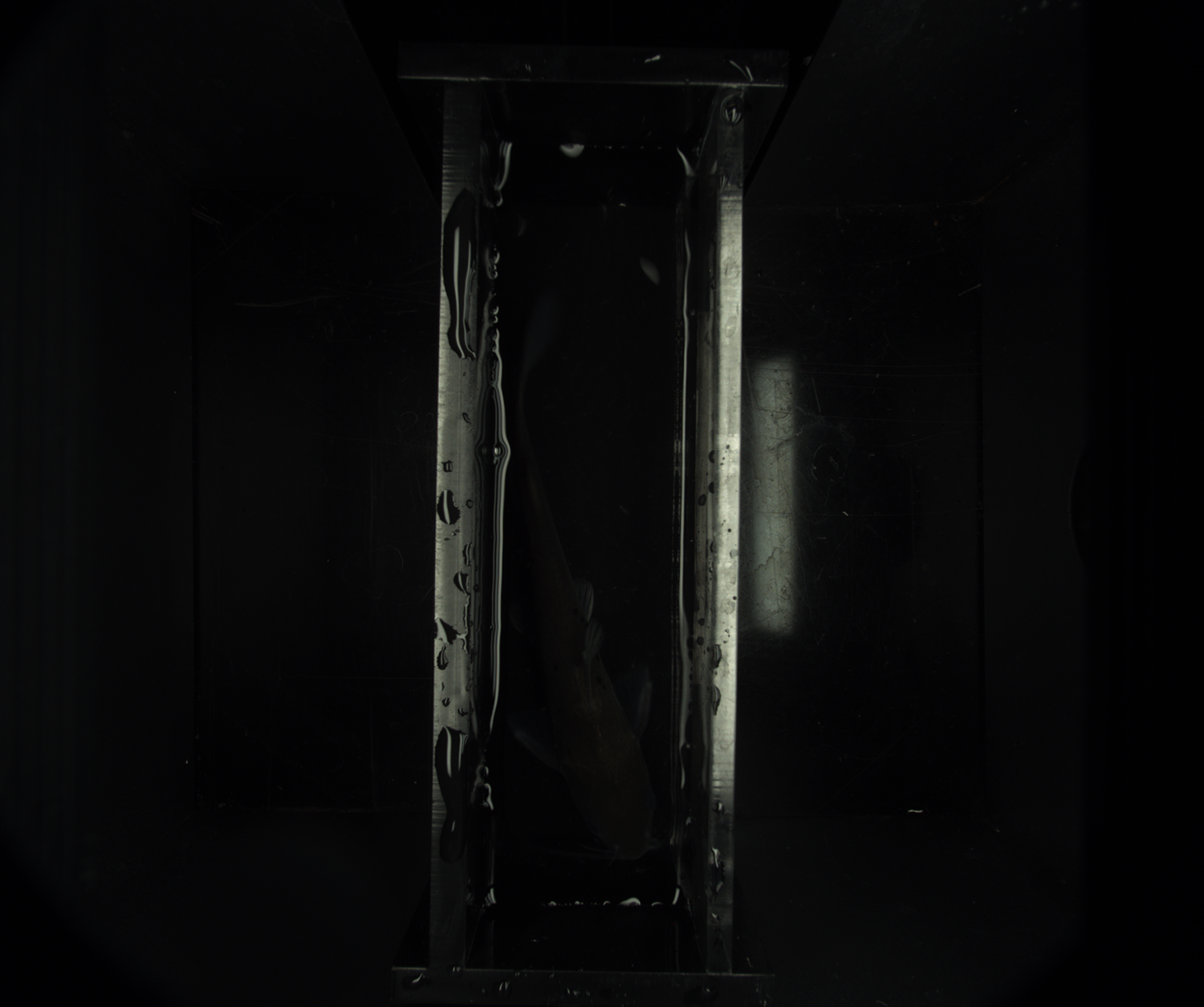

Supplement: S1_Fig — (ZIP) [file pone.0324158.s001.zip › S1_Fig/top view/32.tif]

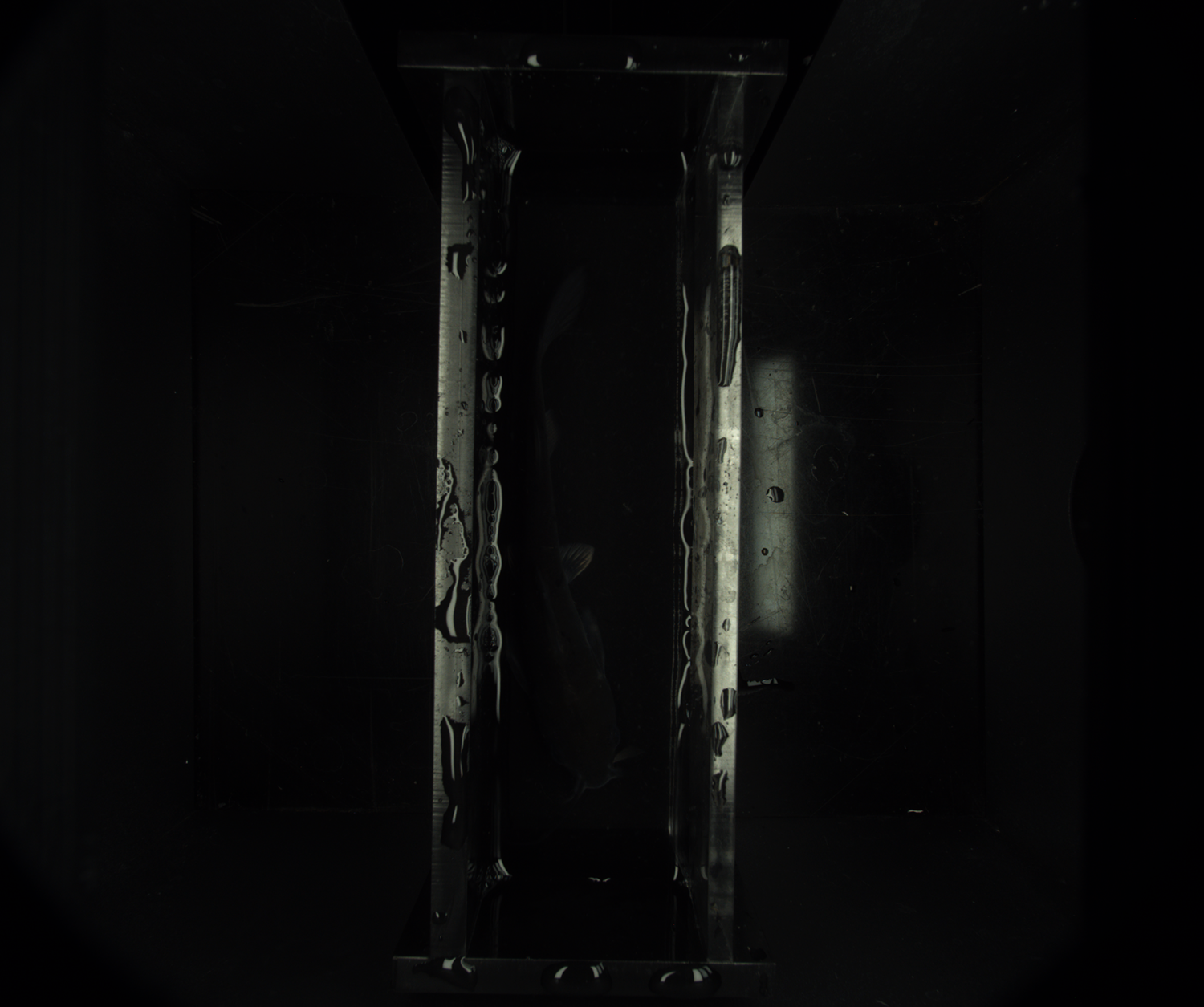

Supplement: S1_Fig — (ZIP) [file pone.0324158.s001.zip › S1_Fig/top view/33.tif]

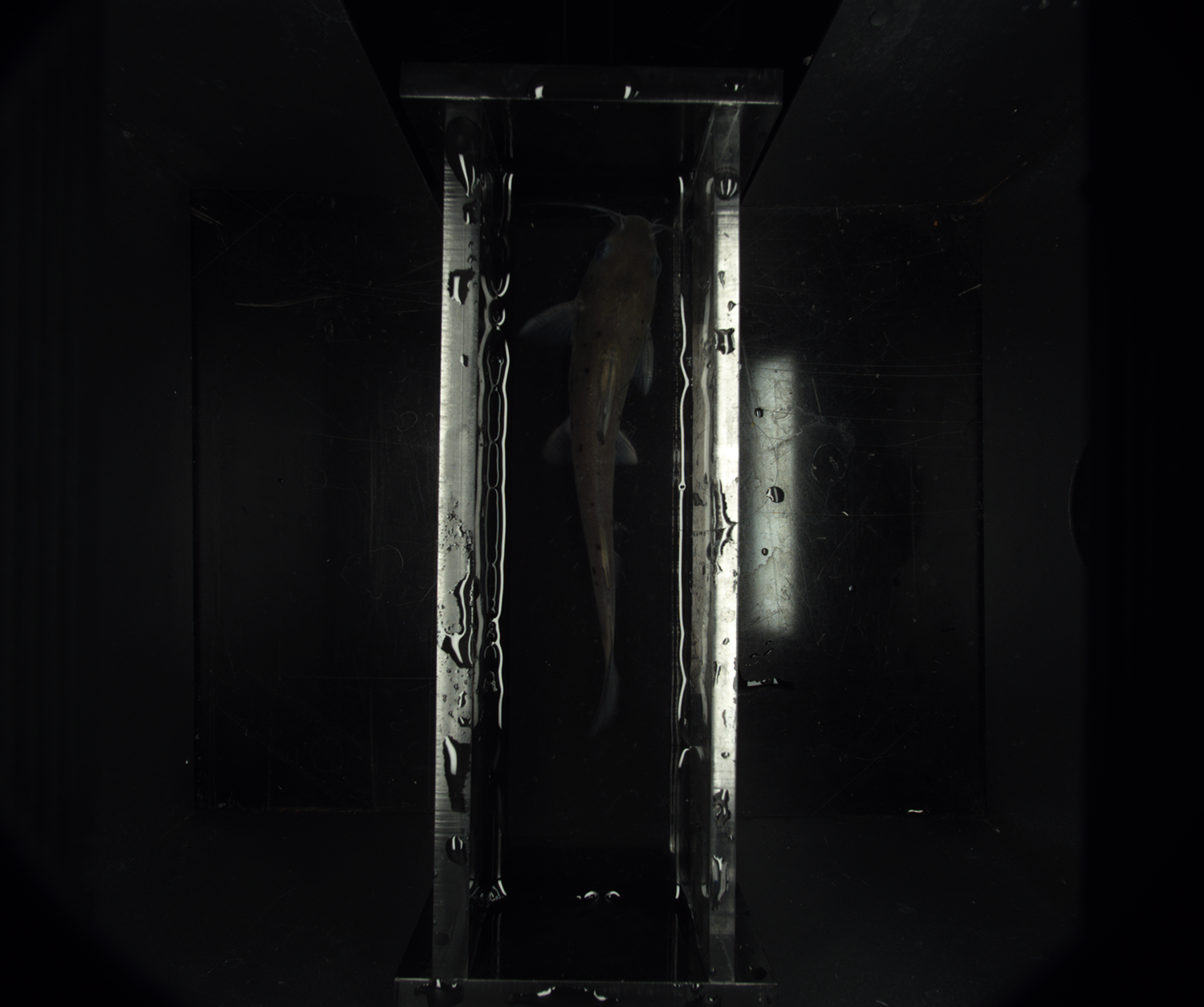

Supplement: S1_Fig — (ZIP) [file pone.0324158.s001.zip › S1_Fig/top view/34.tif]

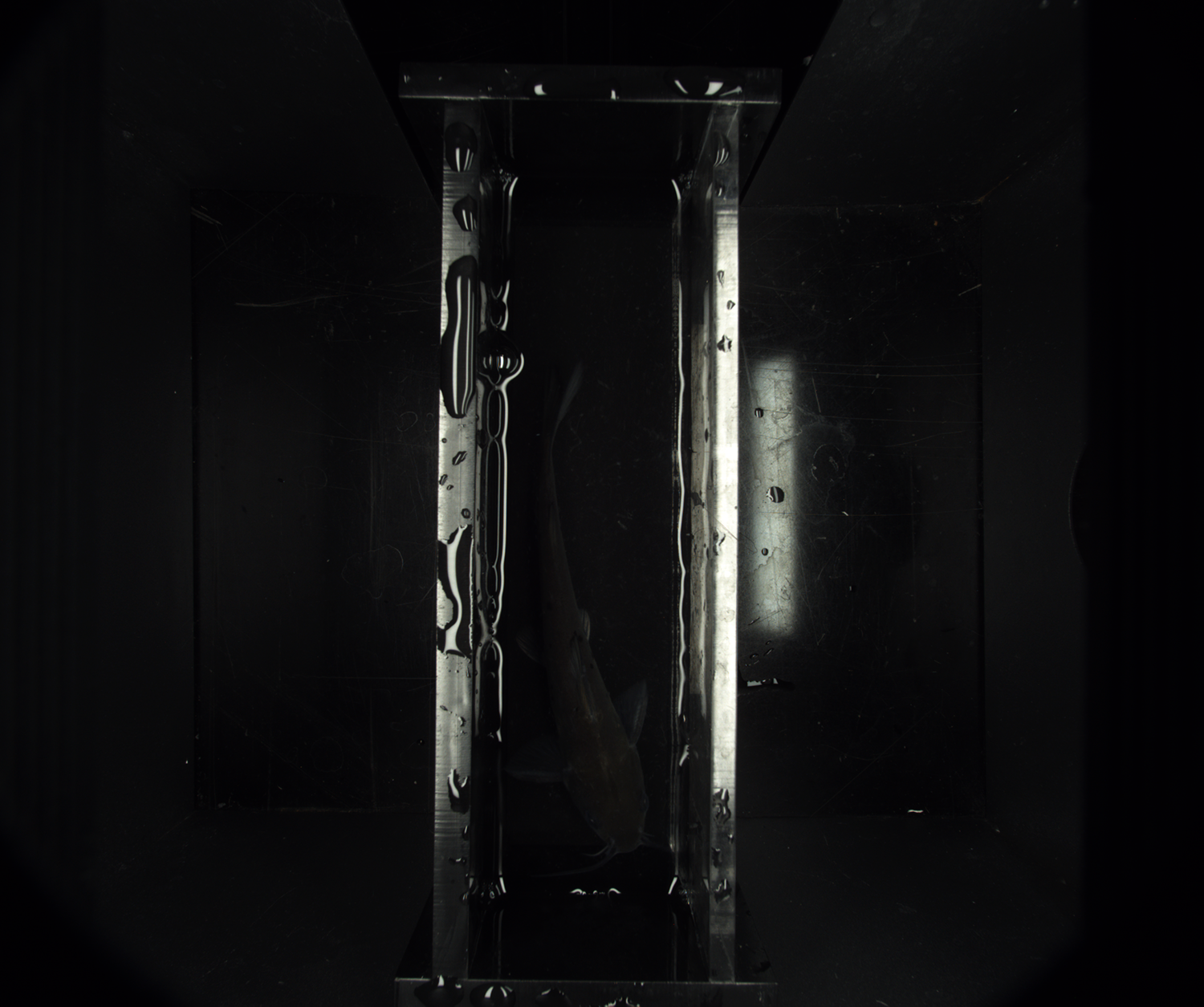

Supplement: S1_Fig — (ZIP) [file pone.0324158.s001.zip › S1_Fig/top view/35.tif]

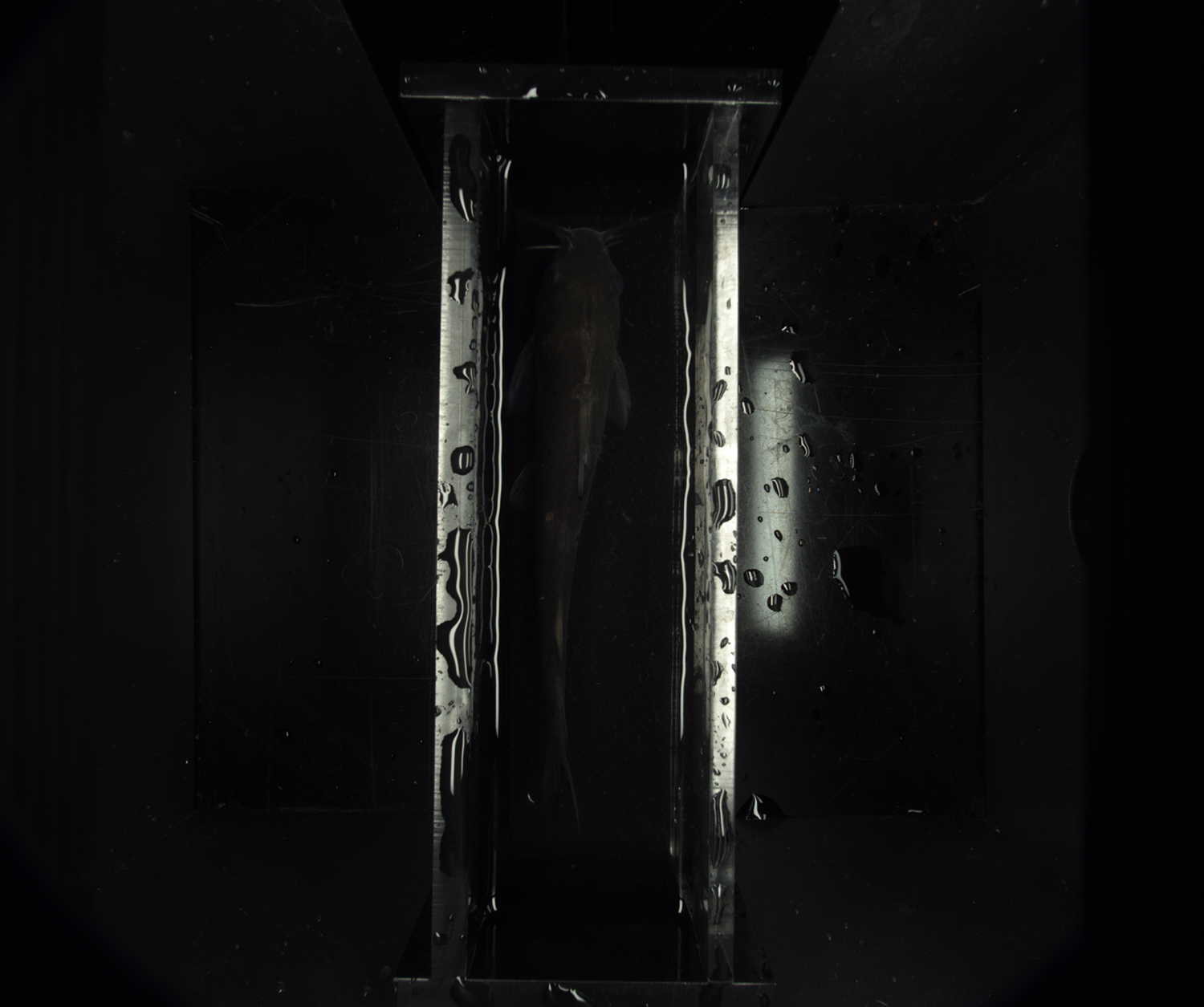

Supplement: S1_Fig — (ZIP) [file pone.0324158.s001.zip › S1_Fig/top view/36.tif]

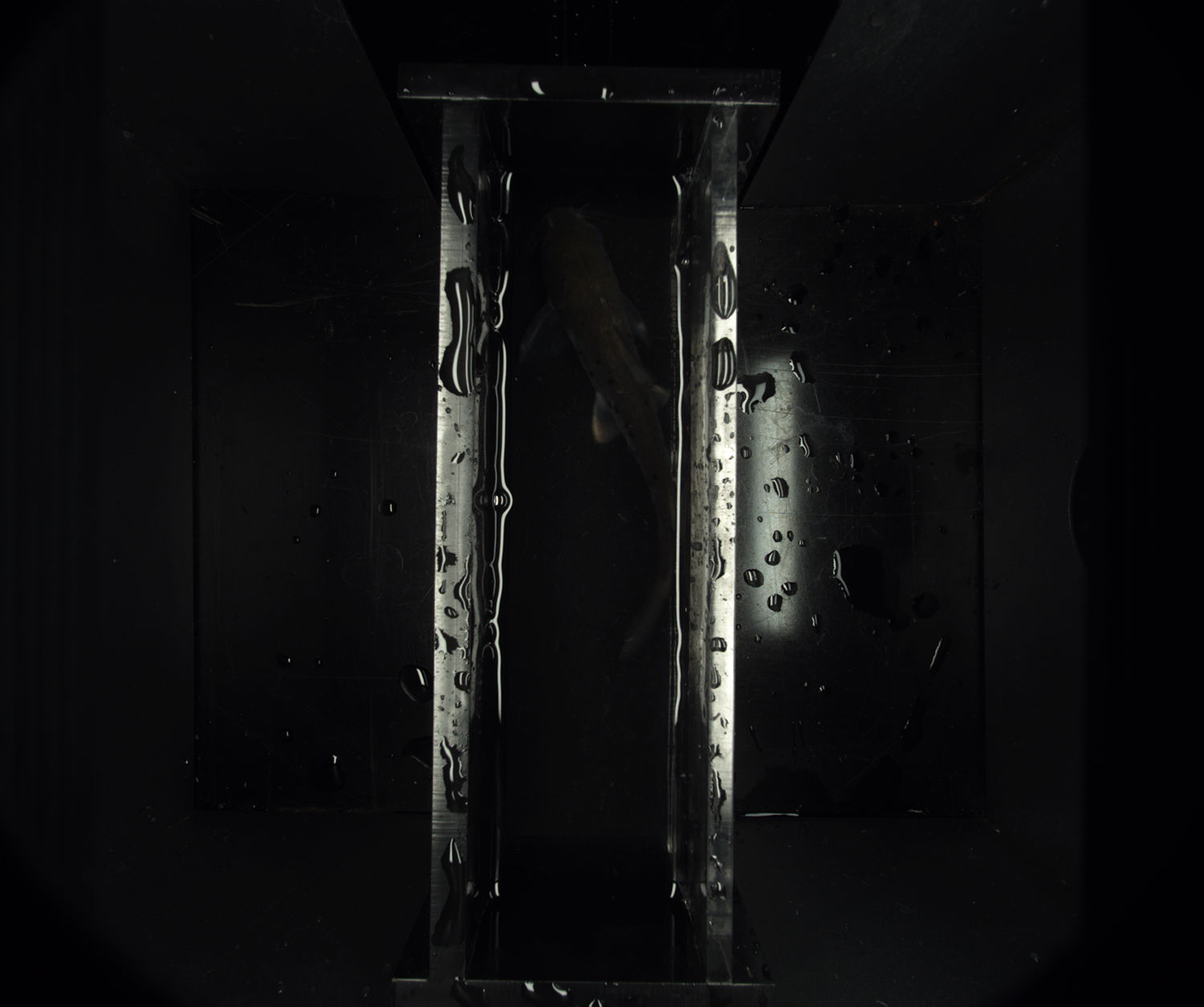

Supplement: S1_Fig — (ZIP) [file pone.0324158.s001.zip › S1_Fig/top view/37.tif]

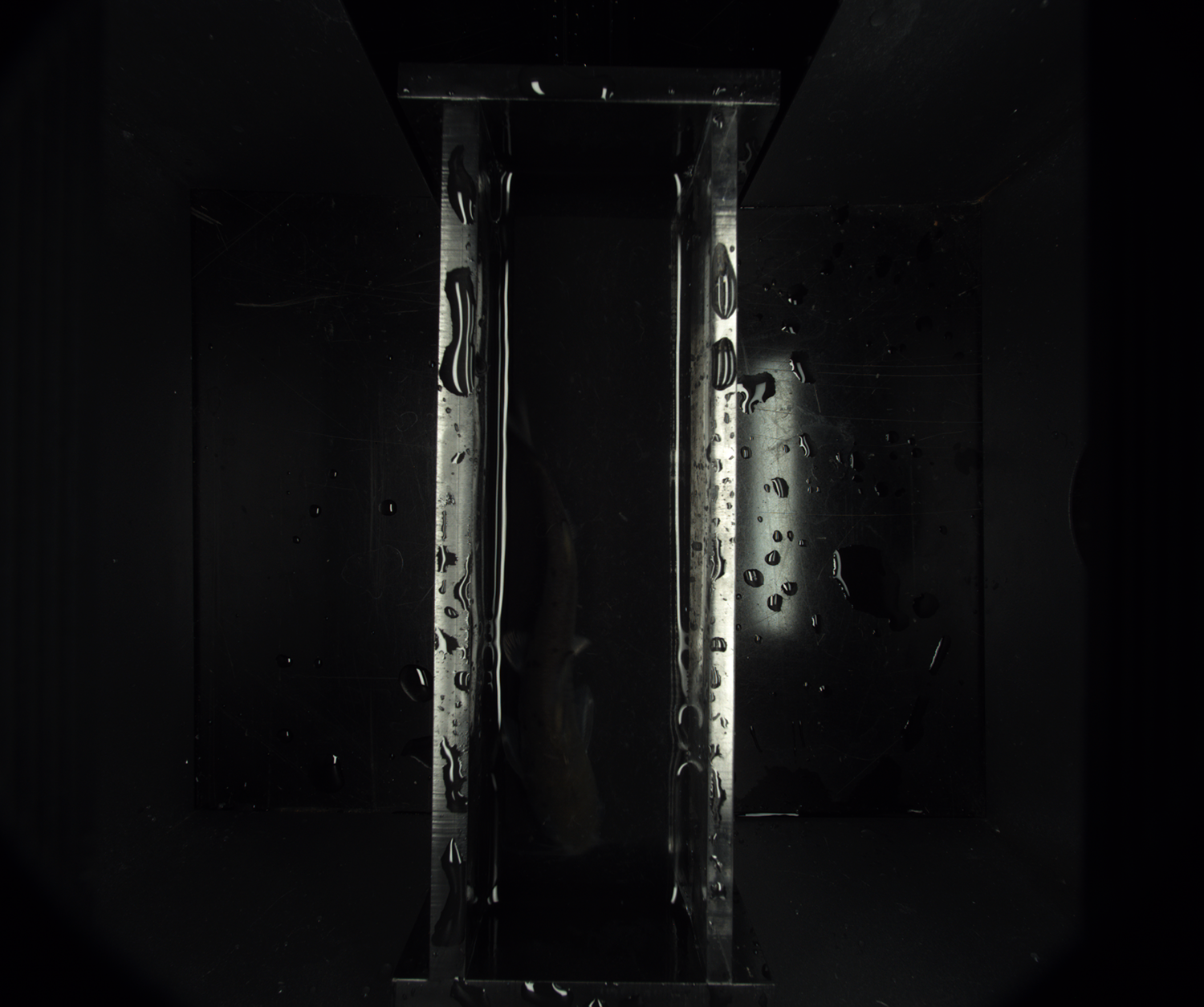

Supplement: S1_Fig — (ZIP) [file pone.0324158.s001.zip › S1_Fig/top view/38.tif]

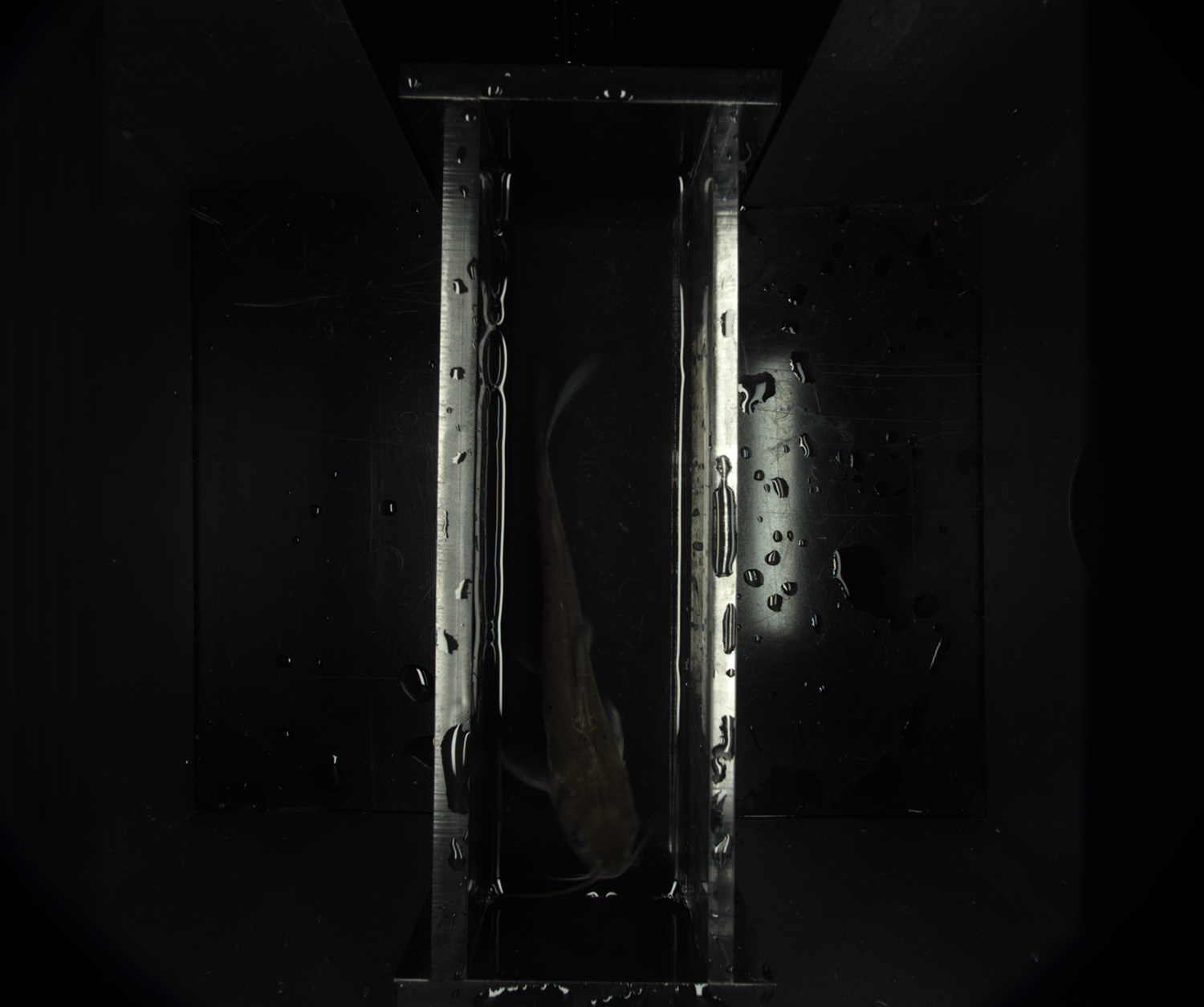

Supplement: S1_Fig — (ZIP) [file pone.0324158.s001.zip › S1_Fig/top view/39.tif]

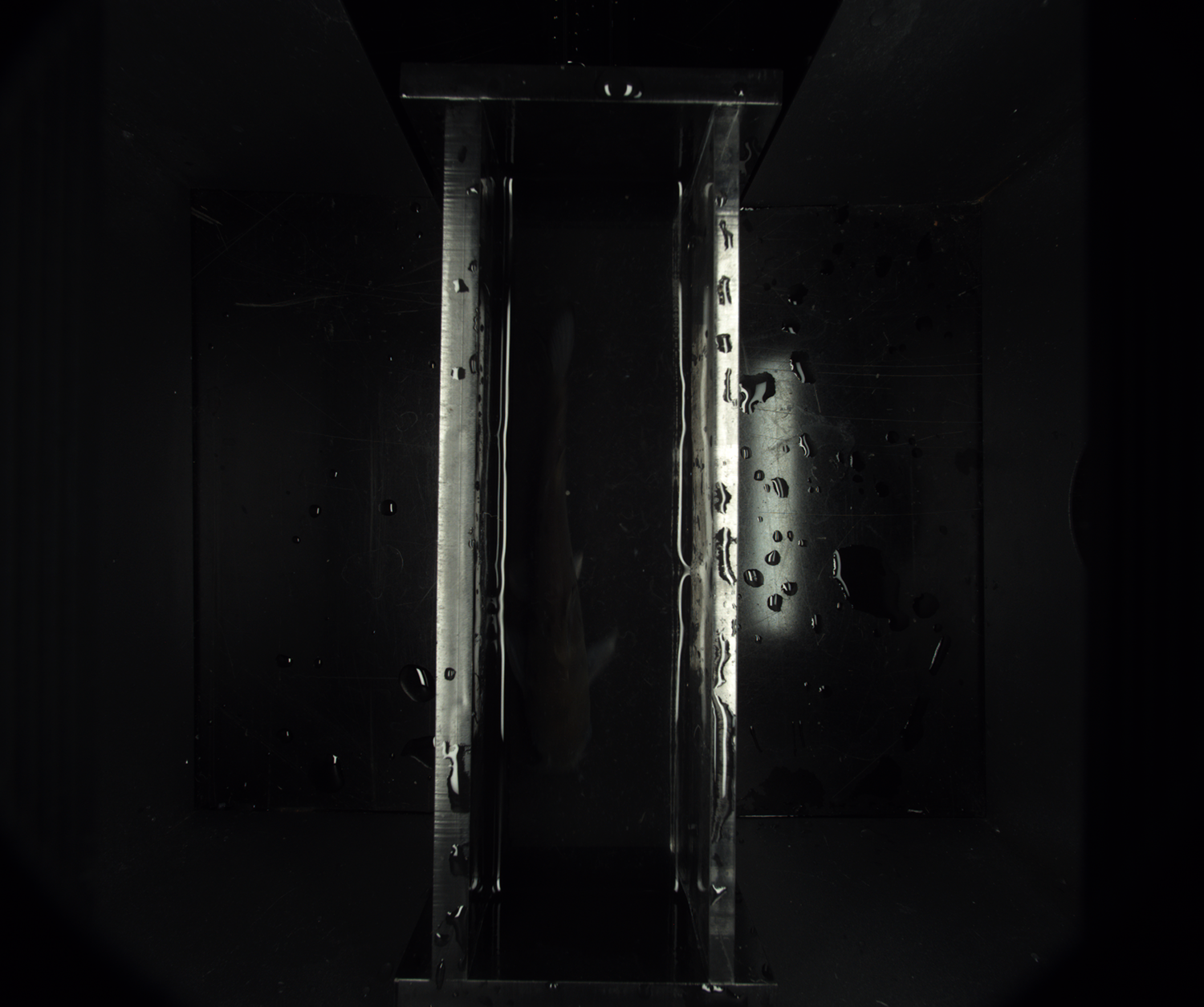

Supplement: S1_Fig — (ZIP) [file pone.0324158.s001.zip › S1_Fig/top view/40.tif]

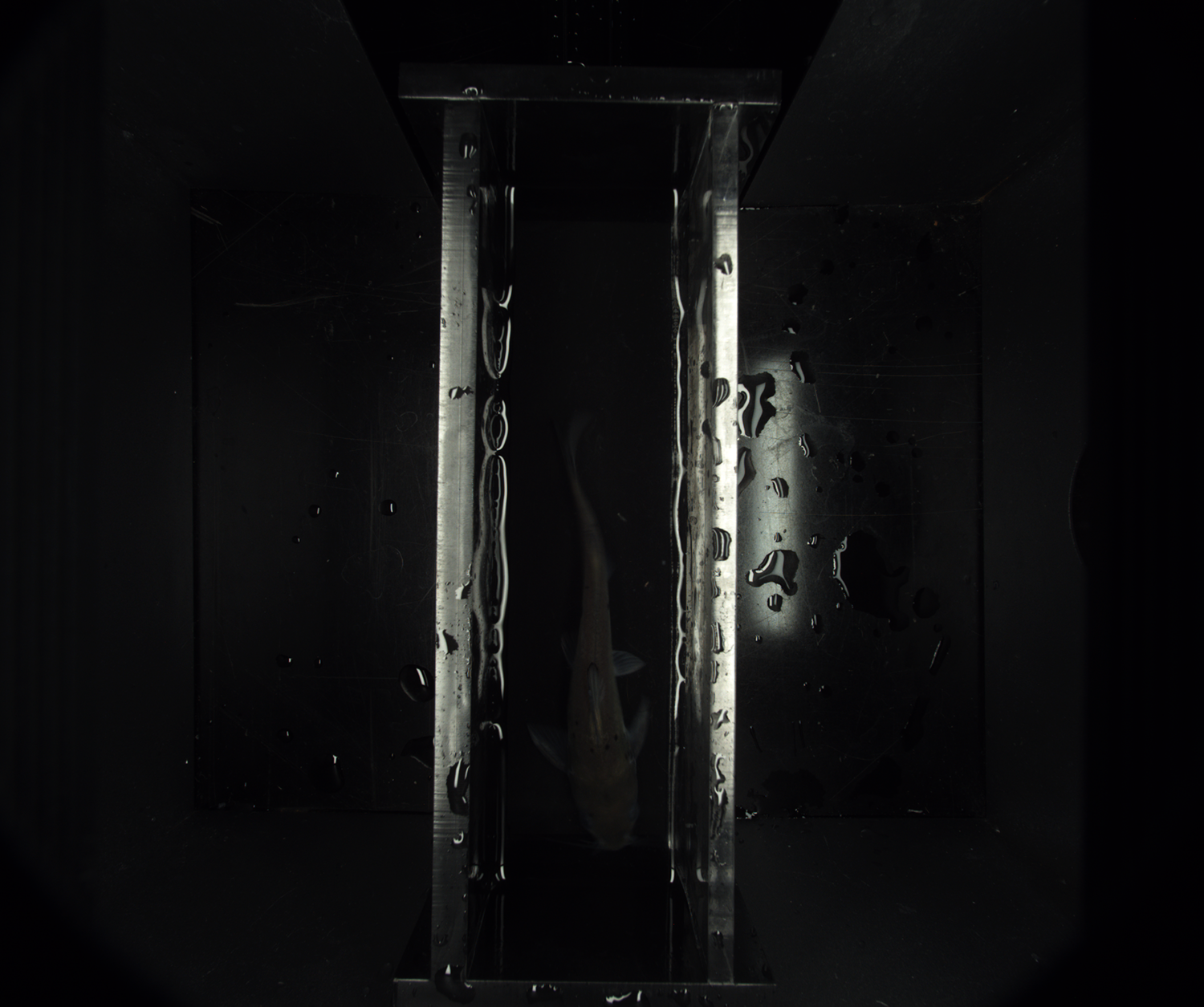

Supplement: S1_Fig — (ZIP) [file pone.0324158.s001.zip › S1_Fig/top view/41.tif]

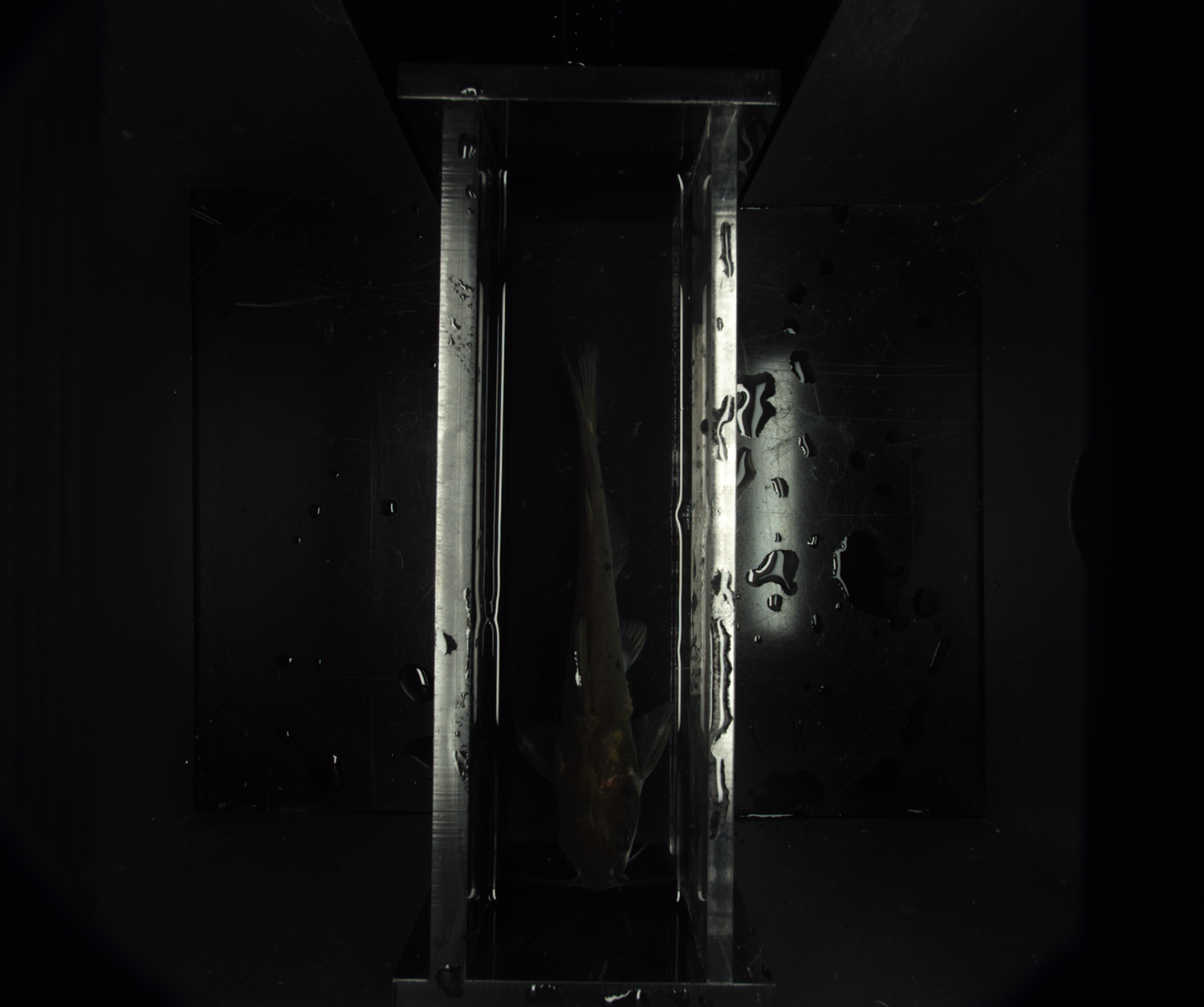

Supplement: S1_Fig — (ZIP) [file pone.0324158.s001.zip › S1_Fig/top view/42.tif]

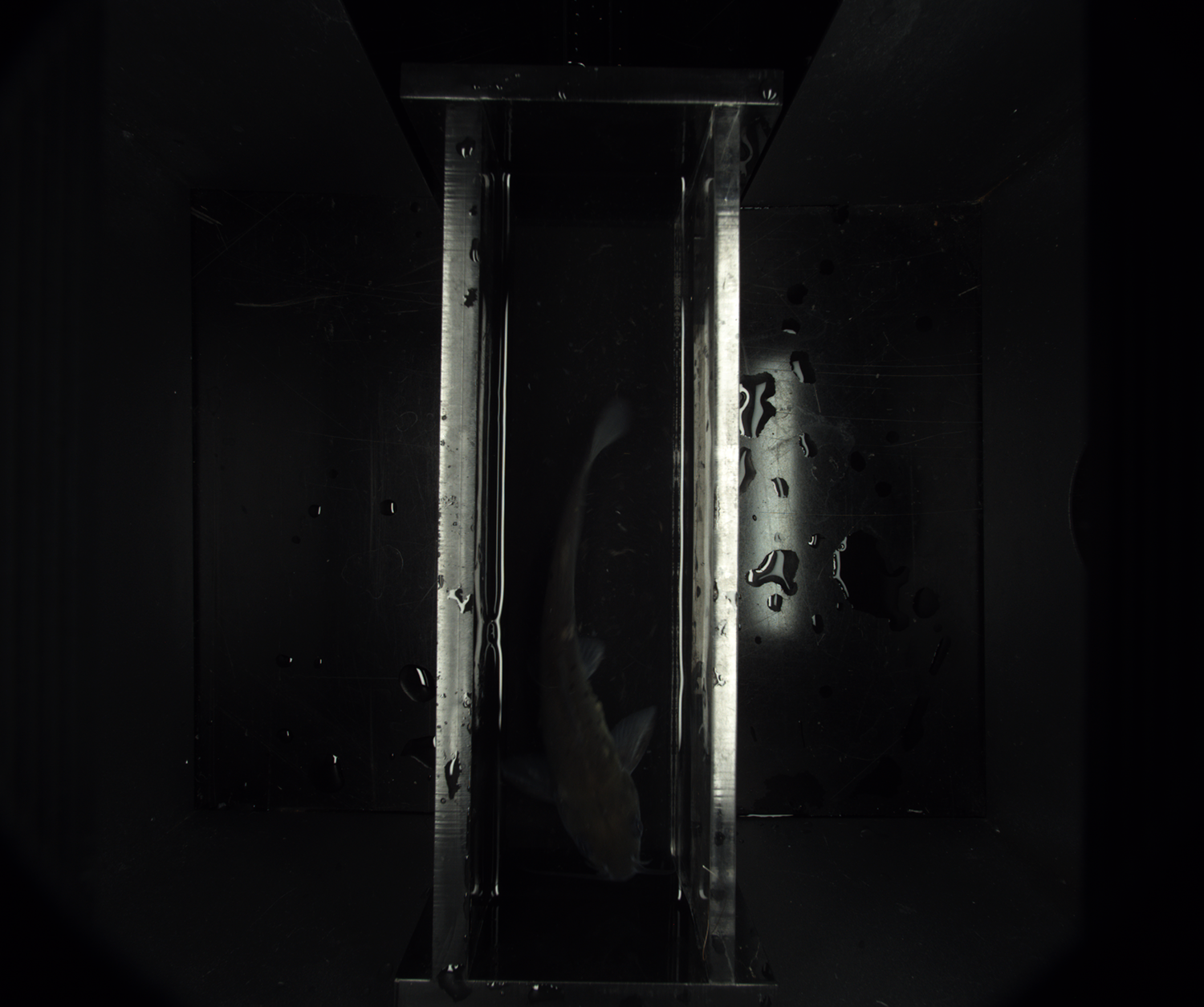

Supplement: S1_Fig — (ZIP) [file pone.0324158.s001.zip › S1_Fig/top view/43.tif]

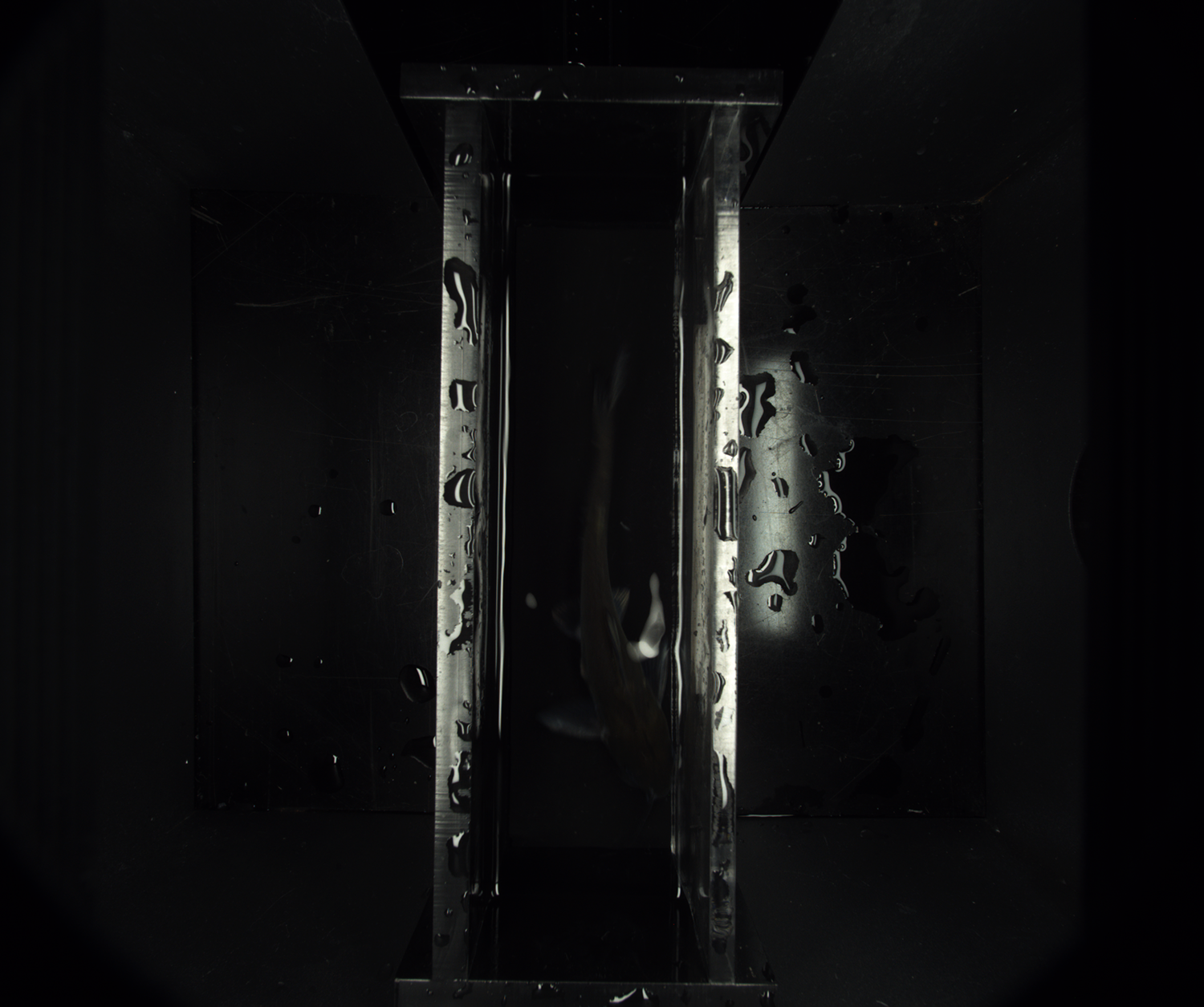

Supplement: S1_Fig — (ZIP) [file pone.0324158.s001.zip › S1_Fig/top view/44.tif]

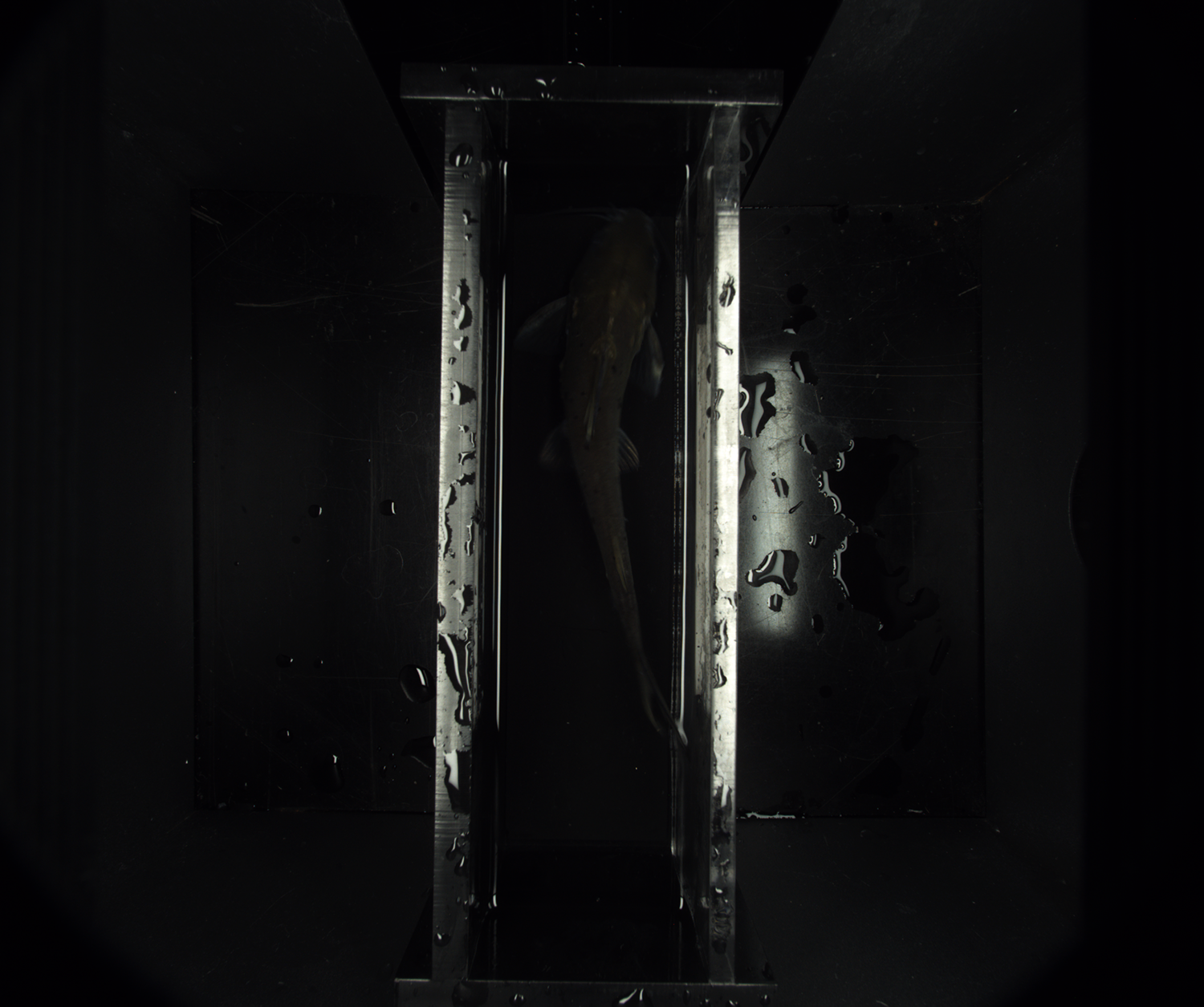

Supplement: S1_Fig — (ZIP) [file pone.0324158.s001.zip › S1_Fig/top view/45.tif]

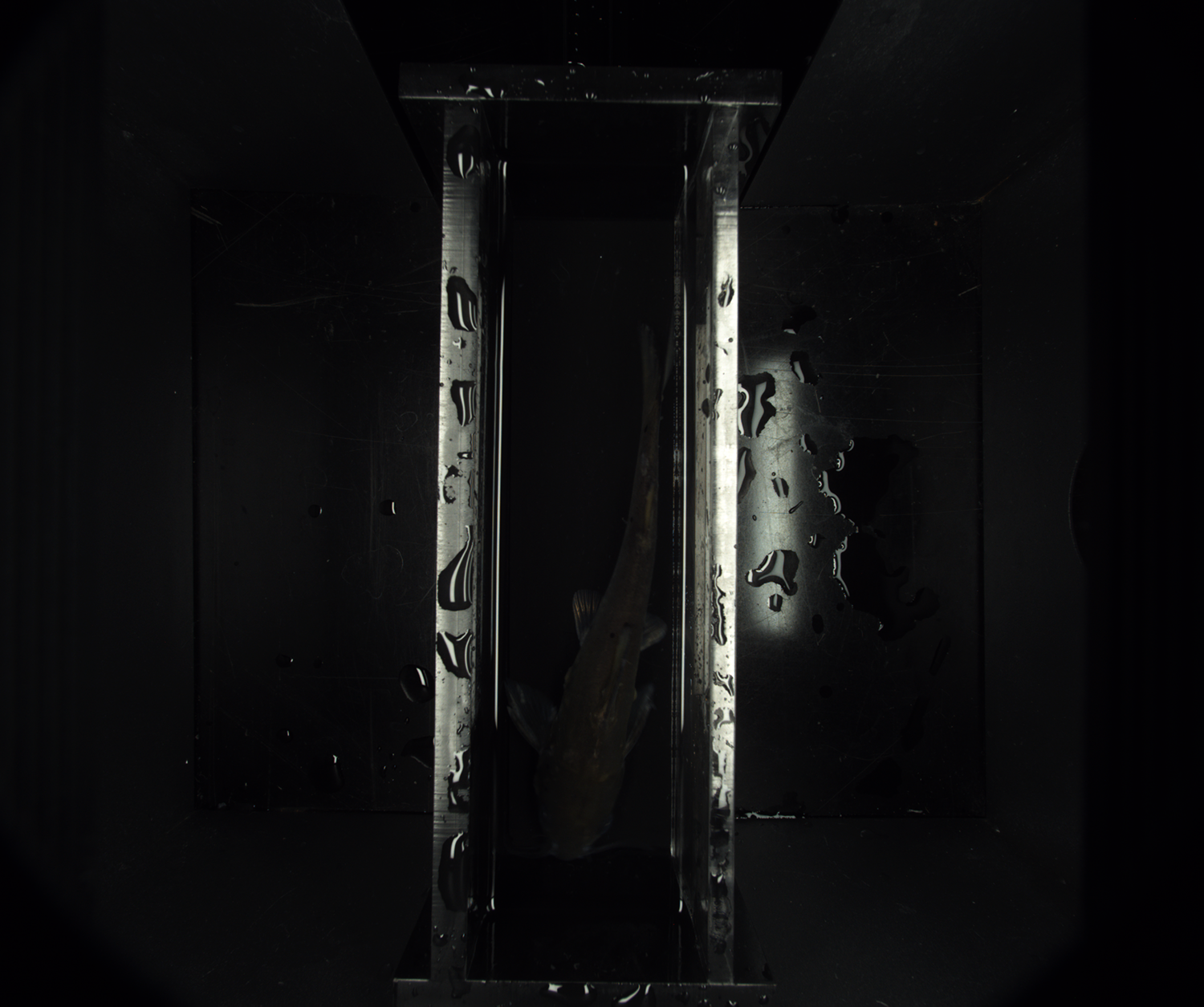

Supplement: S1_Fig — (ZIP) [file pone.0324158.s001.zip › S1_Fig/top view/46.tif]

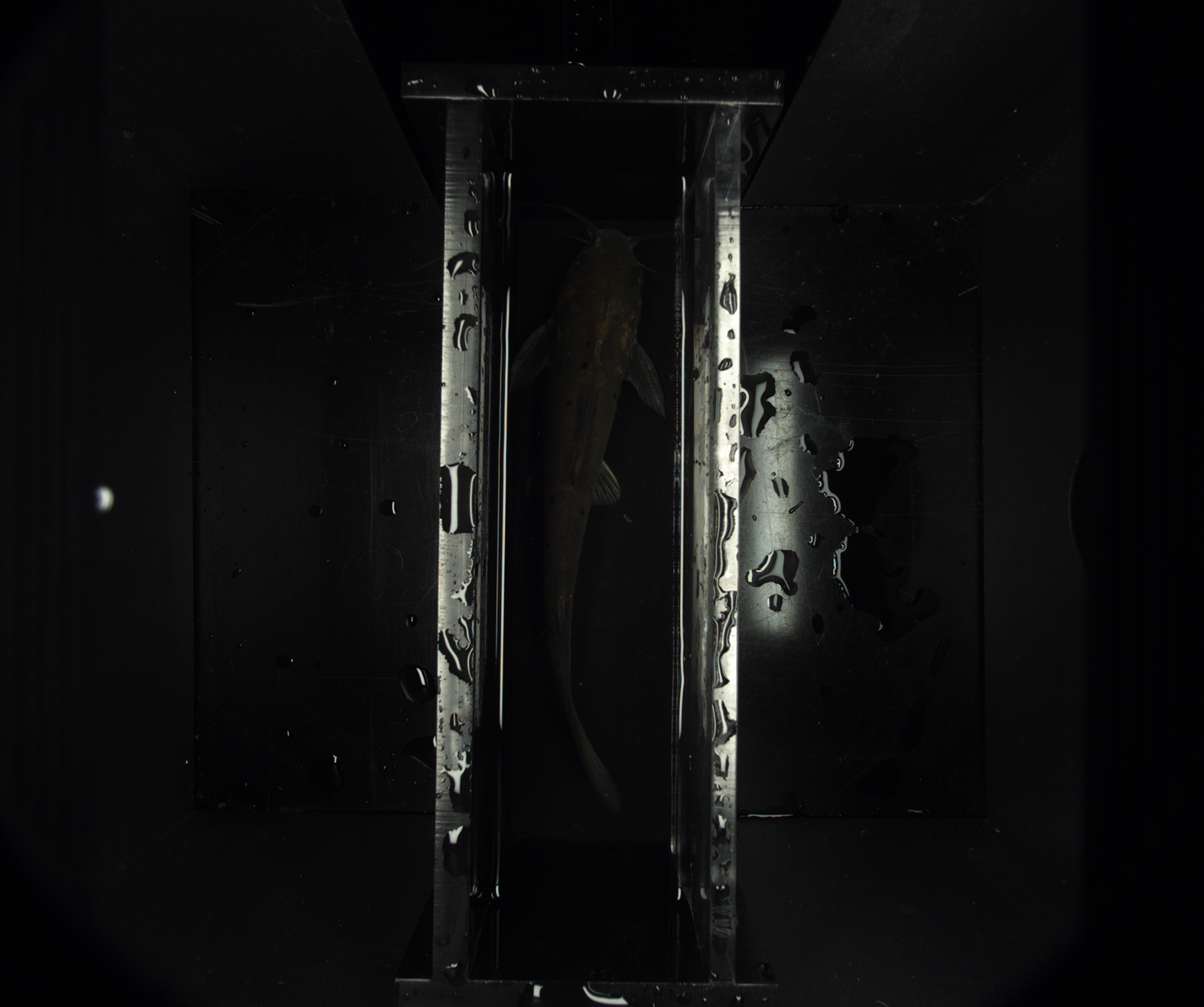

Supplement: S1_Fig — (ZIP) [file pone.0324158.s001.zip › S1_Fig/top view/47.tif]

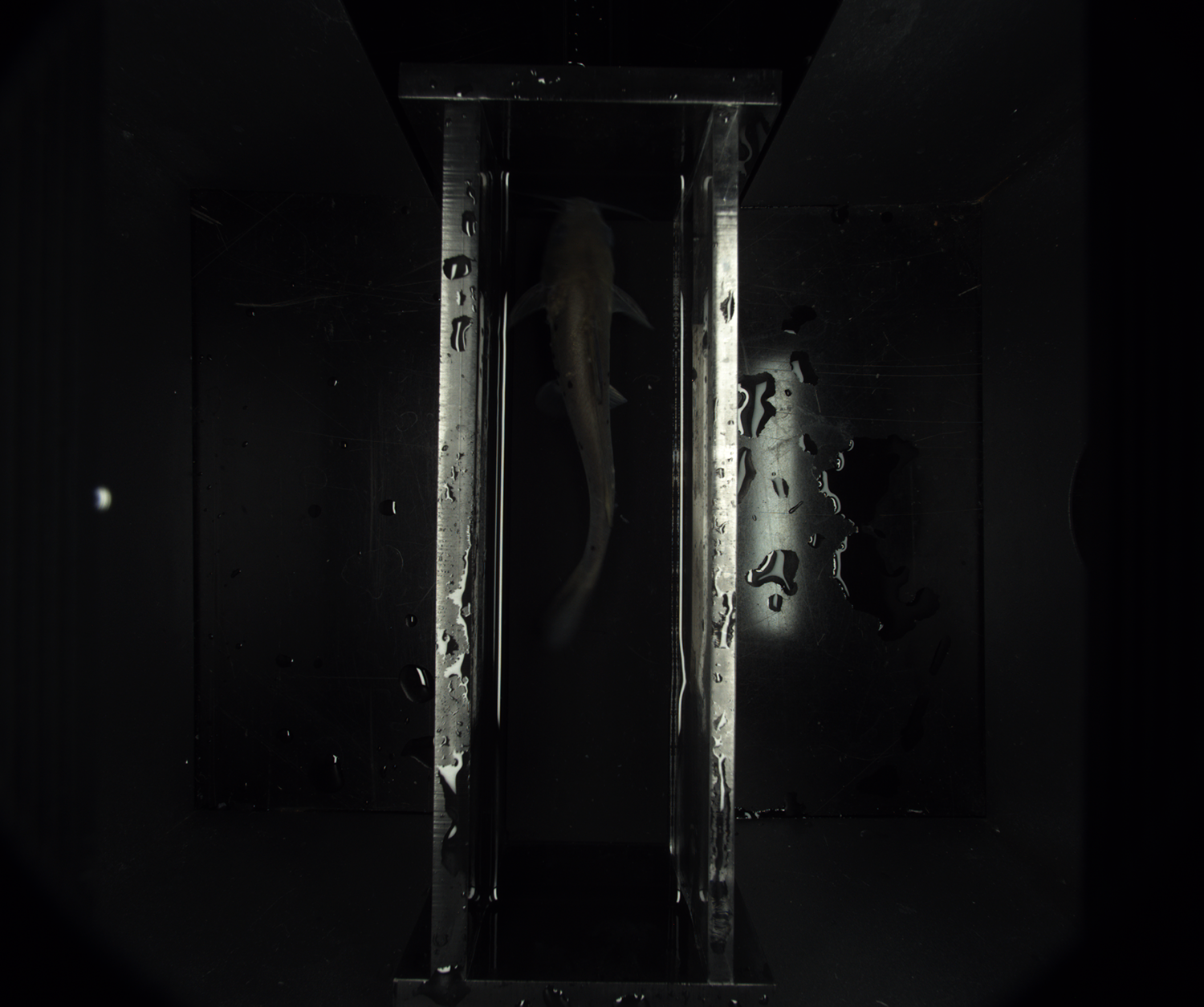

Supplement: S1_Fig — (ZIP) [file pone.0324158.s001.zip › S1_Fig/top view/48.tif]

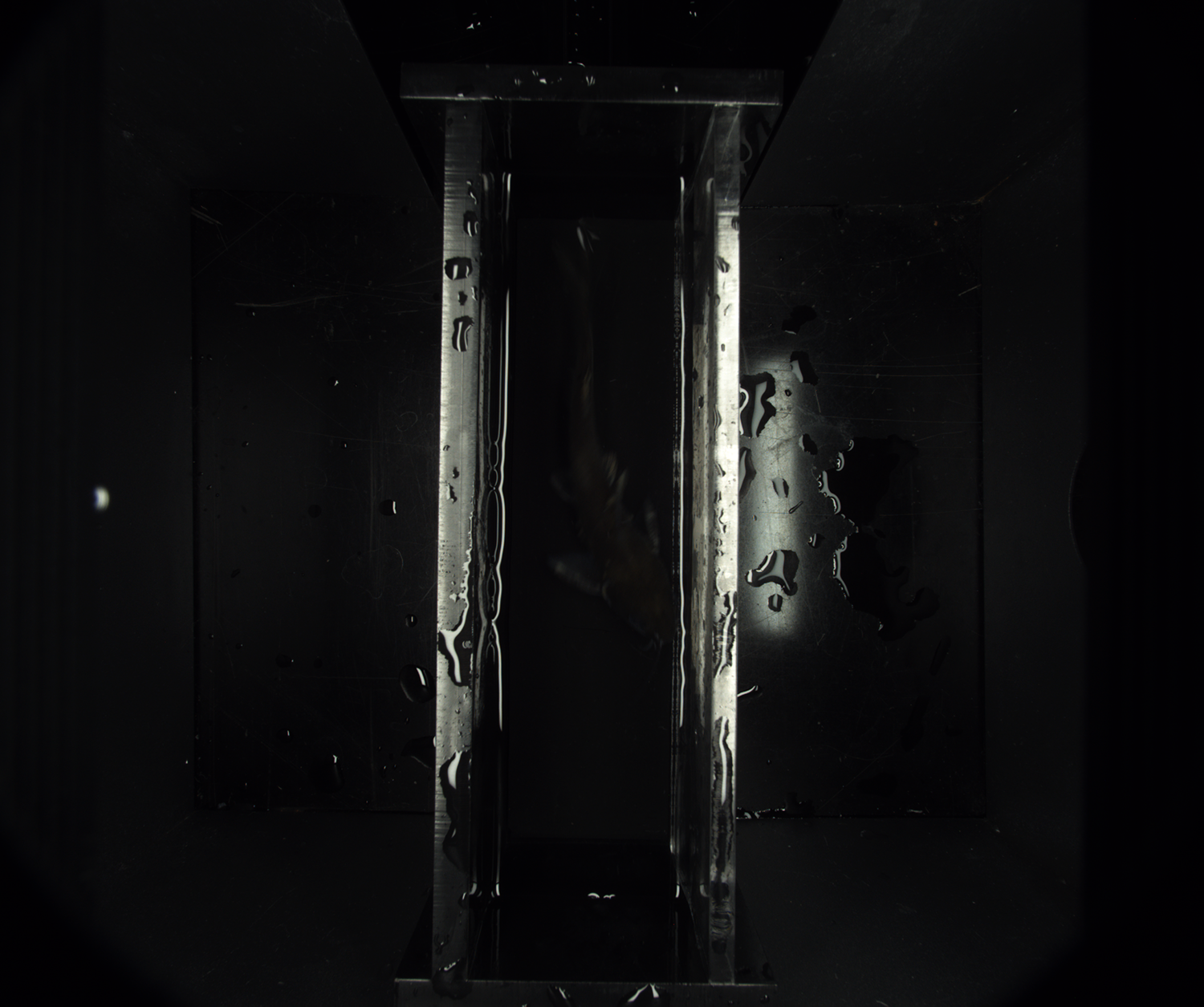

Supplement: S1_Fig — (ZIP) [file pone.0324158.s001.zip › S1_Fig/top view/49.tif]

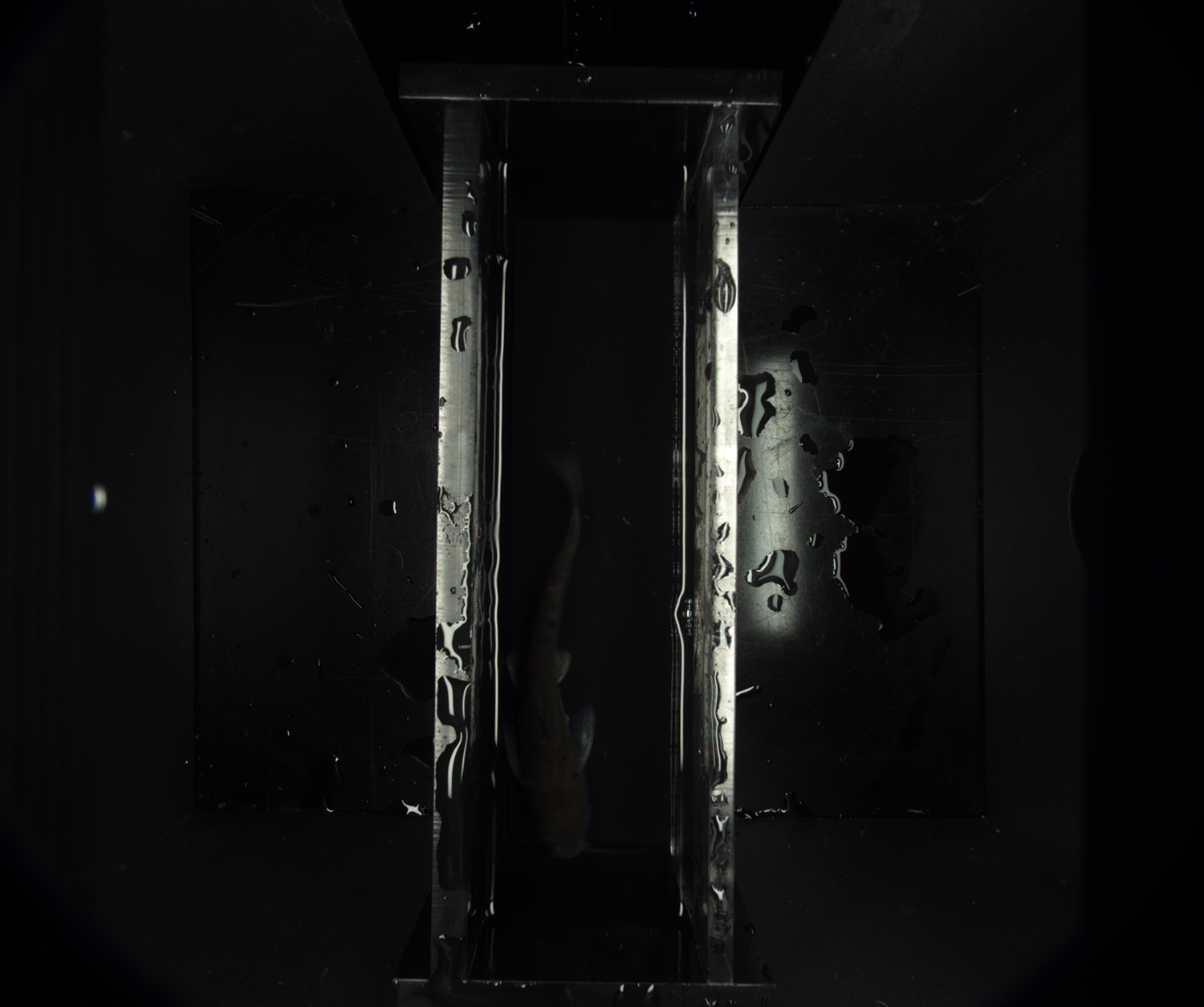

Supplement: S1_Fig — (ZIP) [file pone.0324158.s001.zip › S1_Fig/top view/50.tif]

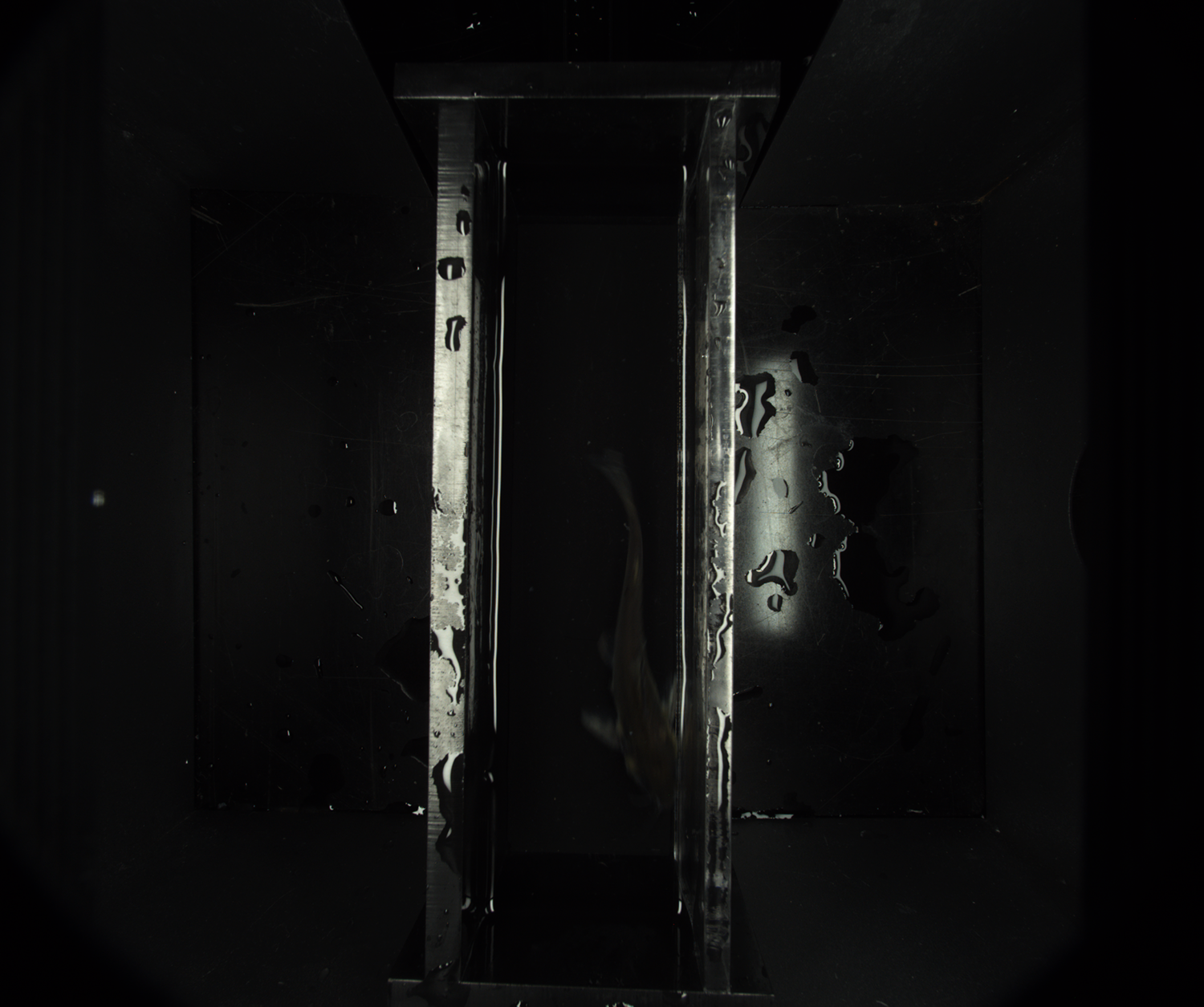

Supplement: S1_Fig — (ZIP) [file pone.0324158.s001.zip › S1_Fig/top view/51.tif]
